# Ultrasonic Surgical Aspirate is a Reliable Source For Culturing Glioblastoma Stem Cells

**DOI:** 10.1038/srep32788

**Published:** 2016-09-08

**Authors:** Jinan Behnan, Biljana Stangeland, Tiziana Langella, Gaetano Finocchiaro, Wayne Murrell, Jan E. Brinchmann

**Affiliations:** 1 Norwegian Center for Stem Cell Research, Department of Immunology, Oslo University Hospital, 1112 Blindern 0317 OSLO, Norway; 2 Department of Molecular Medicine, Institute of Basic Medical Sciences, The Medical Faculty, University of Oslo, Oslo, Norway; 3 Unit of Molecular Neuro-Oncology, Neurological Institute C. Besta, Milan, Italy; 4 Vilhelm Magnus Laboratory of Neurosurgical Research, Institute for Surgical Research, Oslo University Hospital, 0424 Oslo, Norway

## Abstract

Glioma stem cells (GSCs) are thought to be the source of tumor growth and therapy resistance. To understand the biology of GSCs, and target these tumors therapeutically, we need robust strategies for *in vitro* expansion of primary GSCs. To date, tumor core biopsies have been the main established source of GSCs. Since these samples are used for diagnostic purposes, the available tissue for cell culture and therapeutic targeting can be limited. In addition, a core biopsy is usually taken from one part of the tumor, thus would be unlikely to represent intra-tumor heterogeneity. To overcome these problems, tissue fragments from all over the tumor can be collected using an ultrasonic aspirator during surgery, thus assembling a “global tumor biopsy”. Usually, this ultrasonic aspirate (UA) sample is considered as biological waste after operations. Here, we show that UA samples offer a large and reliable source of live cells. Similar to core biopsies, UA samples enriched for GSCs that differentiated into neural lineages, showed inter-individual variation of GSC markers, and induced tumors. Molecular profiling showed that UA samples cover tumor heterogeneity better than core biopsies. These results suggest that UA samples can be used to establish large scale cultures for therapeutic applications.

Gliomas are the most common tumors of the central nervous system (CNS), accounting for around 80% of all malignant brain tumors^1^. According to WHO, gliomas are classified into four main groups (I-IV) based on histological features. Among these, Glioblastoma multiforme (GBM) represents the most common and aggressive primary tumor of the CNS with a median patient survival time of less than 15 months^2^,^3^. Around 90% of the tumors are primary GBMs that arise and develop rapidly in elderly patients mainly without any sign of a previous lesion, while 10% of GBMs are secondary tumors developing from pre-existing lower grade gliomas and are characterized by a younger patient group^4^. GBMs almost always recur after tumor resection followed by chemo- and radio-therapy, often at the site of the initial tumor, but occasionally as far away as the opposite hemisphere^5^,^6^, and the median time to disease recurrence is approximately seven months. It is thought that the highly infiltrative tumor cells and GSCs that escape tumor resection and chemo- and radiotherapy are the reason for the incurable nature of this disease^7^,^8^. Furthermore, it is thought that tumor heterogeneity and development of resistant cell clones play an important role in therapy resistance and tumor recurrence^9^.

Recently, intra-tumoral heterogeneity was described by identifying three different brain tumor types within a single patient using a multi-biopsy strategy^10^. The special intra-tumoral heterogeneity was characterized at molecular level as well^11^,^12^. Clonal and single cell analysis showed that one tumor often contains three subtypes of cells confirming the heterogeneity within GBM^13^,^14^. These studies indicate that a single biopsy would be unlikely to cover the full extent of the intra-tumoral heterogeneity. In addition, biopsy samples could have very limited size and be fully used for diagnostic purposes. This makes the availability of these samples for cell cultures and testing in preclinical and clinical therapeutic settings very difficult sometimes. As cultures of primary GSCs are increasingly being used in the production of GBM vaccines, there is a need for novel and more robust strategies for tumor cell sampling^15^.

One possibility to maximize the yield and heterogeneity of tumor cells could be through the use of ultrasonic aspiration (UA) samples. During GBM operations, an ultrasonic aspirator device is increasingly being used to remove fine fragments of the tumor through torsional oscillation and longitudinal vibration. The irrigated saline solution containing the small tissue fragments is aspirated directly into a sterile bag making a “closed sterile system”, which is considered as biological waste and discarded post-operatively. Some studies have reported the beneficial use of UA samples to increase diagnostic accuracy^16^,^17^.

Recently it was shown that UA samples contain viable tumorigenic cells and can be used as a source for growing GSCs in serum free conditions supplied with EGF and bFGF growth factors^18^,^19^. However, a side-by-side comparison of the tumor core and UA samples has not yet been systematically performed. Therefore, in this work, we compare UA samples to tumor core biopsies for cell yield and viability, phenotype, ability to proliferate under sphere culture conditions, multilineage neuronal differentiation and tumorigenicity. We show that UAs offer an enormous source of cancer cells that can be cultivated, enriched for GSCs, and express a wide range of cancer stem cell (CSC) markers. There are some differences when compared to tumor core samples. Furthermore, we show that multilineage neuronal differentiation, tumorigenicity and the invasion pattern of UA-derived cells seem to be similar to tumor core-derived cells. Thus, UA samples have superior cell yield and cover tumor heterogeneity to a better extent than core biopsies.

## Results

### UA samples offer a greater source of viable cells compared to tumor core biopsies

In this work we have processed 40 UA samples from both low and high grade glioma, and two samples from brain metastasis. A matching tumor core sample was obtained from 18 patients. This information, in addition to patient diagnoses are shown in Table S1.

All samples were processed directly or within a few hours after surgery and small aliquots were sent for bacteriology tests. To estimate the cell yield obtained from UA samples, the tissue fragments within the saline-erythrocyte mixture were spun down, and total tissue and tissue allocated for processing were weighed. After several steps of washing and enzymatic digestion (explained in detail in the attached protocol: Supplementary method), total cell count, viability and cell yield were calculated (Table 1). Viability was comparable between UA and tumor core samples, except in some cases where tumor core contained big necrotic compartments. The obtained cell count from processed tissue was 3-1027 times higher in UA samples than matched tumor cores, and the overall number of obtained viable cells from UA samples was much higher (mean 14 times) than tumor cores (P ≤ 0.0001). The cell yield of viable cells ranged between 4 × 10^6^–95 × 10^6^ per gram of tissue; mean ± SD (29 ± 21 × 10^6^) (Table 1). The RNA integrity number of 11 tested sphere cultures derived from UA samples ranged between 8.4–10 (mean ± SD = 9.3 ± 1.2) and between 6.1–9.4 (mean ± SD = 7.8 ± 0.5) for tumor core biopsies, while it ranged from 4–9.9 (mean ± SD = 7.1 ± 2.4) for UA fresh tissue samples and between 5.8–7.1 (mean ± SD = 7.1 ± 2.4) for fresh core biopsies (Table S2). These data, in addition to negative bacteriology tests, suggest that UA samples offer a large and safe source of viable cancer cells that can be used for cell culture and clinical therapy.

### UA cells can be cultivated in sphere conditions similar to tumor core cells

The sphere culture in serum free medium supplemented with basic fibroblast growth factor (b-FGF) and epidermal growth factor (EGF) is considered as the gold standard culture technique to enrich for GSCs *in vitro* despite a success rate of only around 50%^20^,^21^. To investigate the possibility of establishing sphere culture from UA samples, cells were plated from freshly processed samples directly. Cells from the matched tumor core samples were plated when this material was available.

**Figure 1:**
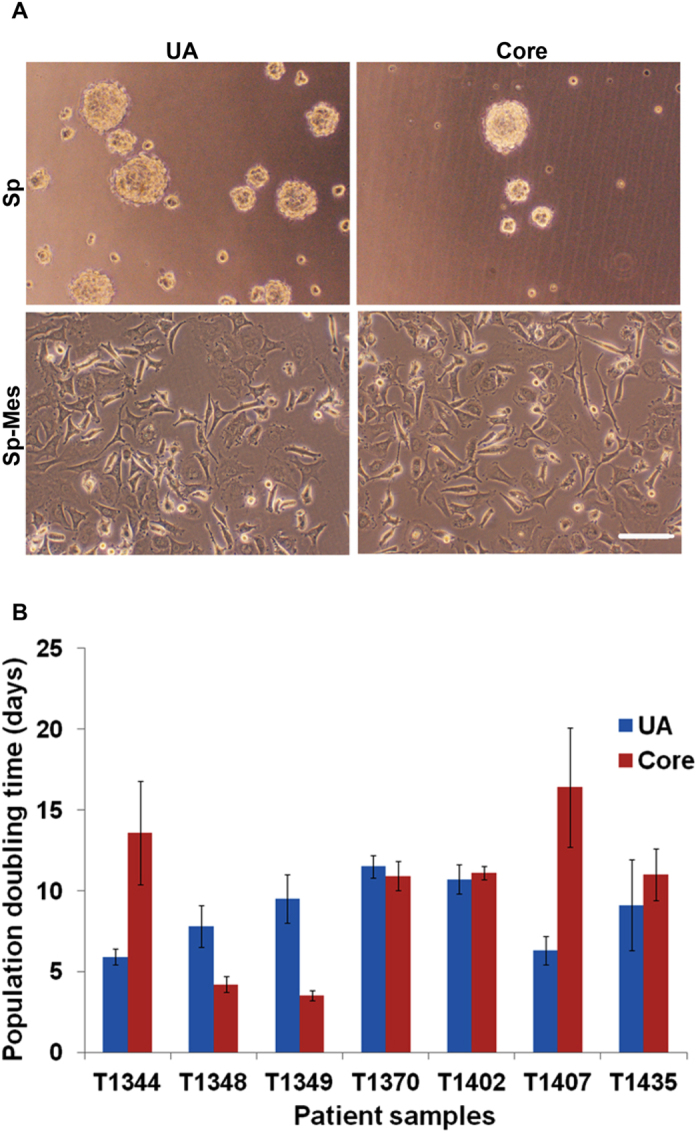
UA-derived cells have similar morphology and growth pattern to cells from tumor core biopsies. (**A**) UA cells and the matched tumor core-derived cells grow in sphere culture conditions in two morphological patterns. Most samples grow as floating spheres, while some grow adherently. Scale bar 250 μM. (**B**) Growth rate of cells derived from UA and tumor core biopsies cultured under sphere forming conditions. Presented cultures are derived from 7 different GBM patient samples and expanded in culture for 3 months. T1348 is giant cell GBM, T1407 and T1435 are recurrent GBM, while the other 4 paired samples are typical primary GBM.

We were able to establish 24 sphere cultures out of 33 (73%) of UA GBM samples including primary, secondary and recurrent GBM. In the group of matched core samples, ten sphere cultures were established out of the 16 matched GBM core samples (63%), while 14 were established from UA samples (88%). Four cases were successfully established from UA samples, but not from core biopsy possibly because of the small cell number obtained from biopsy (Table 1). As previously reported by others, none of our low grade gliomas grew in sphere culture conditions where only a few cells survived in culture after P3, from both tumor core and UA samples (Table S1). Under sphere conditions, out of 23, 16 cultures grew as floating spheres, while the other seven cultures enriched for cells that grew adherently. Tumor core was available for two of those seven cultures and had similar growth patterns (Fig. 1A, Table S1).

Cells in sphere culture continued to proliferate exponentially and cultures were passaged every 10–14 days (Fig. 1B). Cultures that are used in this work were less than passage 11 (P11), and CGH-analysis showed that cells enriched in sphere cultures shared genomic aberrations with their original parental tumors^22^. Thus UA cells can successfully grow in sphere conditions, in the current study with greater efficiency than cells from tumor core biopsies.

### Molecular profiling using global mRNA analysis shows that UA samples and core biopsies show differential expression of several cancer pathways

**Figure 2:**
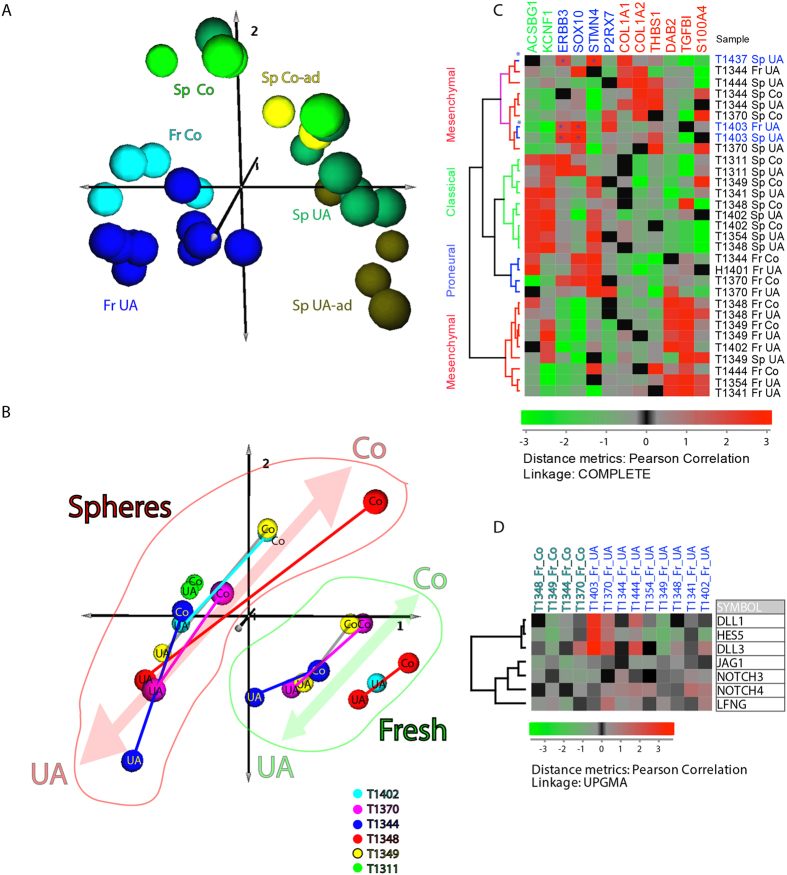
Gene expression profiling of the UA and Co samples. (**A**) Global profiling of gene expression. To visualize similarities or dissimilarities between Core (Co) and Ultrasonic aspirate (UA) samples, we used principal component analysis (PCA) of gene expression. In this 3D PCA plot each individual sample is represented with a ball of a certain colour. Principal components PC1, PC2 and PC3 were represented as axes 1, 2 and 3. While the PC1 separated spheres (right) from the fresh tissues (left) the second PC separated fresh tissue UA samples (dark blue) from the Fresh tissue Co samples (light blue) and the sphere (Sp) cultures expanded from UAs (darker shades of green) from the Sp cultures expanded from the core tissues (lighter shades of green). This was the case both for so-called “adherent” spheres and the spheres grown as “floating spheres”^22^. (**B**) Global PCA analysis according to the patient annotations. Individual sets of UA and Co samples were matched as they originated from the same patient. This was the case both for spheres (Sp) and Fresh tissues (Fr). The samples originating from core samples (Co) were clearly separated (pointing towards “north-east” from the samples originating from the ultrasound aspirates (UAs) (pointing towards “south-west”). This was the case both for fresh samples (right quadrants) and spheres (left quadrants). Each ball represents one sample. Each colour represents one patient. (**C**) Hierarchical clustering (HCL) plot showing subtyping of the sphere and fresh cultures according to the 12 gene signature^22^. Included in this analysis are: a) the new samples from this study (GSE84707) and b) several UA samples from the previous analysis^22^. The dendrogram colours red, blue and green specify mesenchymal, neural/proneural and classical subtypes, respectively as previously determined^22^. The expression values were log_2_ transformed. (**D**) HCL plot showing differential expression of the notch signalling pathway genes. Dendrogram colours light and dark blue specify core and UA samples respectively. The expression values were log_2_ transformed.

To visualize the differences and compare UA to core biopsy samples we used principal component analysis (PCA). Using PCA to visualize global gene expression we could detect constellations such as: Fresh tissue (Fr) cluster (represented in two shades of blue) and Sphere culture (Sp) cluster (represented in different shades of green) that could be clearly separated from one another by the first principal component on the 3D PCA plot (Fig. 2A). Furthermore, the fresh tissue core samples (light blue) were grouped apart from the fresh UA tissue samples (dark blue) indicating significant differences in the gene expression. Also the sphere samples generated from either the UAs or the tissue cores could be separated by the second principal component on the 3D PCA plot. In conclusion, the PC1 separated spheres from fresh tissues while the PC2 roughly separated UA samples from Core-samples.

To further analyze differences between core samples (Co) and UA samples we used PCA based on global analysis of six individual patient sets. The patients that were not represented with both, Co and UA samples, were excluded from this analysis. Similarly to results presented in (Fig. 2A), the first PC component predominantly separated the fresh tissues from spheres (Fig. 2B). For clarity these two groups were outlined as the two assemblies: red (cluster of sphere samples to the left) and green (cluster of fresh tissues to the right) on the 3D PCA plot (Fig. 2B). The second PC component further separated samples roughly to UA samples and Co samples. This was visualized on the 3D PCA plot as a segregation towards “north-east” for Co samples and towards “south-west” for UA within each individual assembly (Fig. 2B). In conclusion, PCA analysis showed the existence of clear differences in gene expression between UA samples and Co samples both at each group level (eg. Comparing “all cores to all UAs”) and at the individual patient level (eg. comparing Cores and UAs from the same patient to one another).

To check whether there are differences in the subtypes of UA samples and their matched core biopsies, we subtyped GBM tissue samples and sphere cultures according to our previously determined set of 12 genes^22^. The subtypes of new samples that are described here (GSE84707) showed matching subtypes (between UA and Co samples) in all cases except for two patients: one fresh tissue (T1344, UA is Mesenchymal, while Co is of Proneural subtype), and one sphere culture (T1349, where UA was Mesenchymal, whilst Co was of Classical subtype) (Fig. 2C). Combining our small sample set with TCGA did not affect the subtype of paired UA and core biopsies (Supplementary Figure S1). However, three samples (T1437 Sp-UA, T1403 Fr-UA/Sp-UA) that expressed both Proneural and Mesenchymal genes, were classified as Proneural when our set was fused with TCGA (Fig. 2C and Supplementary Figure S1).

To identify dysregulated signalling pathways and differentially expressed genes (DEGs) between the two groups we used fold-change analysis. Hierarchical clustering was performed on Fr-Co (fresh tissue of core biopsy) and Fr-UA (fresh tissue of UA) samples in order to define the set of most variable genes (Supplementary Figure S2). Firstly, we identified 352 differentially expressed genes (DEGs) (Supplementary Figure S2) and also identified several pathways that were differentially regulated in UA and Co samples (Supplementary Table S3) such as notch (Fig. 2D and Supplementary Figure S3), p53 (Supplementary Figures S4 and S5) and ECL interaction pathways (Supplementary Figures S6 and S7). HCL analysis showed that several key players of the notch pathway were up-regulated in the UA samples (Fig. 2D). Also several other cancer related genes such as proto-oncogenes jun and fos were upregulated in UA samples (Supplementary Figure S8). Thus, UA samples seem to feature an increased expression of a variety of cancer-related pathways and genes. The gene expression profiling of the UA and the Co samples indicates that the UA method may be better at capturing the tumorigenic signature of the biopsies.

### Targeted DNA-mutational sequencing shows that UA samples contain some mutations that are not detected in tumor core biopsies and cover tumor heterogeneity more than core biopsies

**Figure 3:**
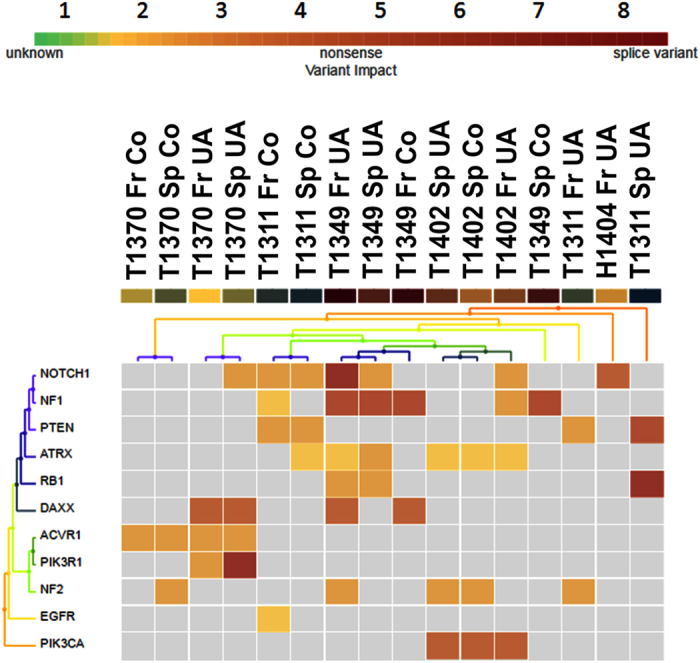
Targeted NGS analysis showing that UA samples harbor more mutations than core biopsies in most sequenced samples. Sequencing analysis was performed on the target sequenced genes. Amplicon sequences were aligned to the human reference genome GRCh37 (hg19) in the target region of our custom gene panel using Torrent Suite™ software 5.0. SNVs calling was performed using the Variant Caller (v5.0.4.0). Three primary GBM and one secondary GBM, in addition to one normal sample (epileptic patient) were inlcuded in this analysis. Most common mutations were NF1, NOTCH1, ATRX and NF2, and more mutations were detected in UA samples that core biopsies. However, core biopsies expressed some mutations that were absent in UA samples (not more than one mutation usually). In one case, secondary GBM (T1311), core biopsy expressed more mutations than the UA. The legend at the top of the heat map is color-coded for the following variant impacts using the associated score values (unknown: 0, synonymous: 1, missense: 2, non-frameshift block substitution: 3 non-frameshift insertion: 4,nonsense: 5, stop-loss: 6, frameshift block substitution: 7, frameshift insertion: 7, frameshift deletion: 7, splice variant: 8.

To check for the differences between UA and core biopsy samples on a mutational level, we performed targeted next generation sequencing (NGS). Our panel included 29 genes that were frequently reported to be mutant in adult human gliomas, pediatric gliomas and neurofibromatoses or schwannomatosis (Supplementary Table S4). We found variation in the mutational load among patients. most common mutations were (NF1, NOTCH1, ATRX, NF2). Within single patients, comparing UA to core biopsy from fresh tissue and cultured cells, there were 1-4 mutations shared among those samples, while many mutations were expressed in UA samples and absent in core biopsies, none or only one mutation was expressed in core samples and absent in UA samples (Fig. 3) and (Supplementary Table S5). For example, targeted NGS analysis of the patient T1349 show that fresh UA sample contains more mutations than core biopsy, four mutations were detected in UA sample, but not in the core biopsy. Also sphere culture derived from this sample expressed three of those four mutations, while the one derived from the core biopsy expressed only the common mutation in this patient (NF1). In total, three out of four patients that were sequenced, expressed more mutations in their UA sample compared to their core biopsy. Only the tumor core of secondary GBM (T1311) expressed more mutations than UA counterpart and the sphere culture derived from this core biopsy expressed more mutations than the one derived from the UA sample (Fig. 3) and (Supplementary Table S5).

Our targeted NGS analysis indicated that UA samples contain higher mutational load in three out of four patients and many mutations could not be detected in tumor core biopsies, thus molecular profiling depending on a single biopsy cannot be certain to represent the entire tumor heterogeneity of a GBM and in most analyzed cases, UA samples capture a wider spectrum of tumor heterogeneity than tumor core biopsies.

### Individual variability but no significant difference in expression of stemness, neuroal and proliferation markers between UA and tumor core cells

**Figure 4:**
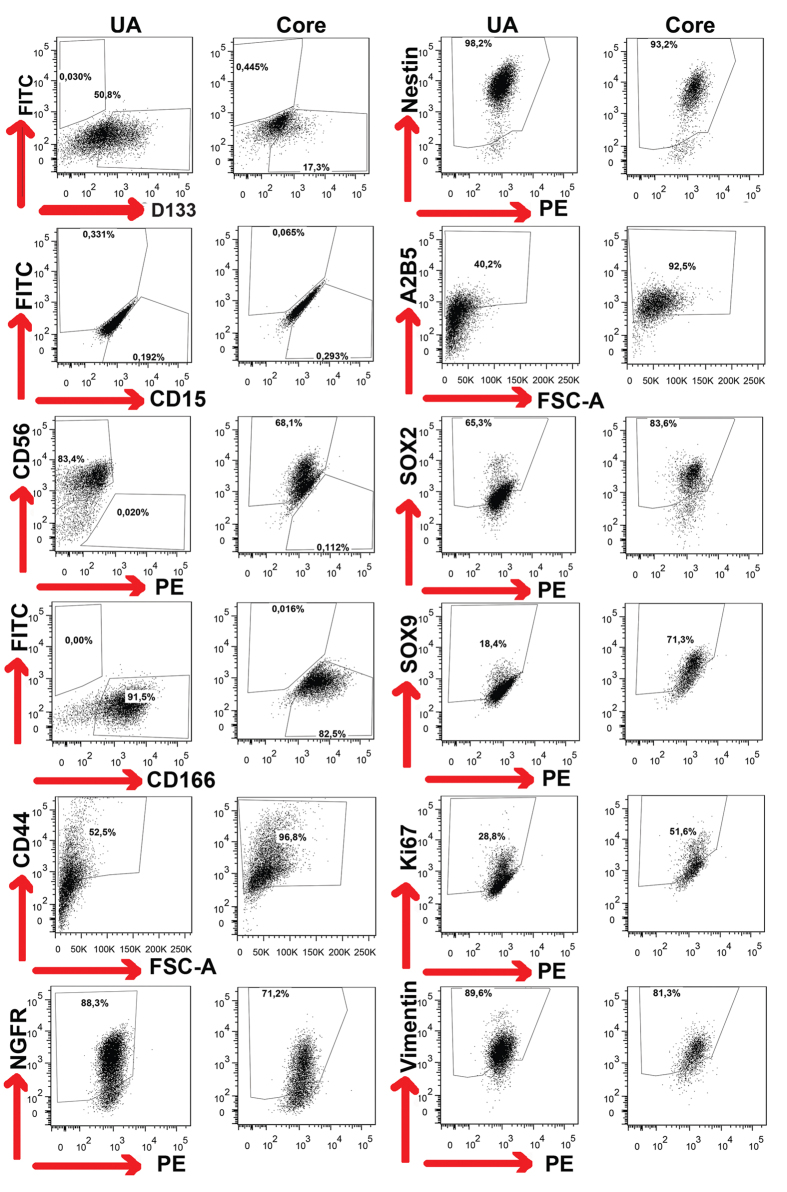
UA cells express varying levels of GSC, neuronal and proliferation markers compared with those of tumor core-derived cells. FACS analysis showing the difference in marker expression between UA-derived cells (UA) and tumor core-derived cells (Core). See Table S5 for marker expression in all patients. The FACS gate was set on total population after excluding cell debris and doublets using the Fluorescence Minus One Control strategy.

To assess the protein expression of stemness and proliferation markers, we performed flow cytometry analysis on cells enriched under sphere cultures from both UA and tumor core samples. We used 26 surface and intracellular markers that were previously reported for GSCs, NSCs and proliferation (Supplementary Table S6). The expression of CD15, SOX2, SOX9, YKL40 and the proliferation marker Ki67 tended to be higher in cells derived from tumor core compared to UA cells, but the difference was not statistically significant. On the other hand, the expression of CD166, CD133, CD44, A2B5, CD56, NGFR and DCX seemed to be higher in UA cells, but also not statistically significant, while the expression of CD9, Nestin, GFAP, CD24, PDGFRb, PDGFRa, MAP2, TUBIII, S100 were consistently high in both UA and tumor core samples (Fig. 4) and (Supplementary Table S6). However, the expression of CD133 and CD15, the best known GSC markers, were absent or very low in around 50% of the sphere cultures. Thus, cells derived from both UA and tumor core biopsy under sphere culture conditions express glioma stemness markers and proliferation markers with some but not significant variability.

### UA cells have multilineage neural differentiation potential similar to tumor core cells

**Figure 5:**
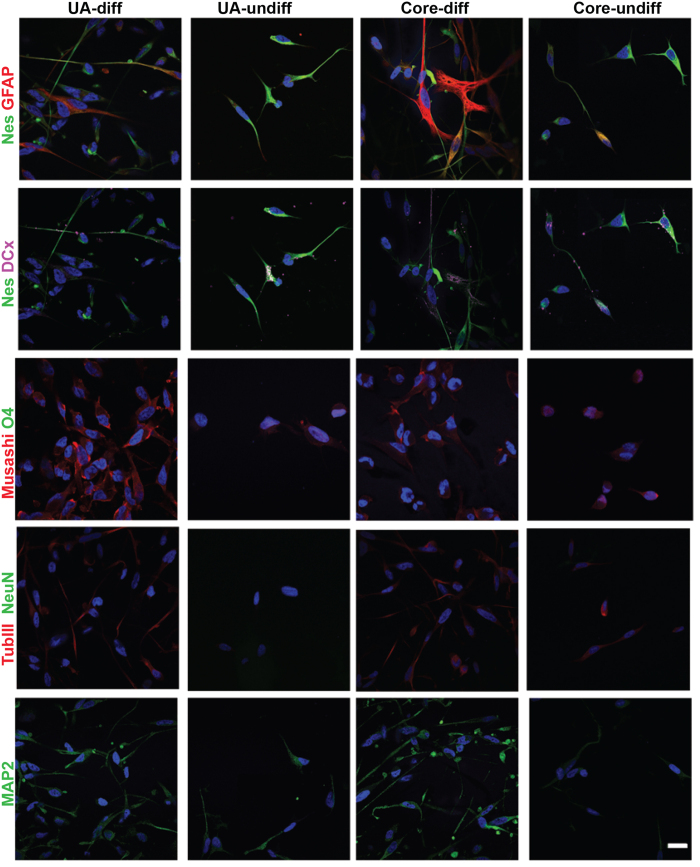
UA cells have multilineage neural differentiation potential similar to tumor core-derived cells. Staining for neuronal, glial and oligodendrocytes markers after two weeks of differentiation for UA cells (first column) and tumor core-derived cells (third column) show that differentiated cells from both samples, UA and Core, express higher levels of GFAP, BIII and down regulate the expression of immature neural stem cell markers such as Nestin and DCx than undifferentiated control cells. The same laser intensity and confocal settings were used for the comparison. Scale bar: 20 μM.

To examine the neural differentiation potential of UA cells, we subjected them to a neural differentiation protocol for two weeks in the presence of serum, B27 and vitamin A and the absence of growth factors. Under neural differentiation conditions, cells from sphere cultures of both UA and tumor core samples gave rise to cells that expressed markers of mature neurons and glial cells (MAP2, β-TubIII and GFAP), but not oligdendrocytes (O4). However, the differentiated cells did not lose the expression of immature neural/neural stem cell markers (Musashi, DCX and Nestin), but they had reduced expression level of DCX and Nestin compared to undifferentiated cells (Fig. 5). The same laser intensity and confocal settings were used for UA and the matched core samples compared to their undifferentiated cells. This assay was done on samples from five patients. Thus, sphere culture cells from both UA and tumor core biopsies have similar multilineage neural differentiation capabilities.

### UA cells have tumorigenic potential similar to tumor core cells

**Figure 6:**
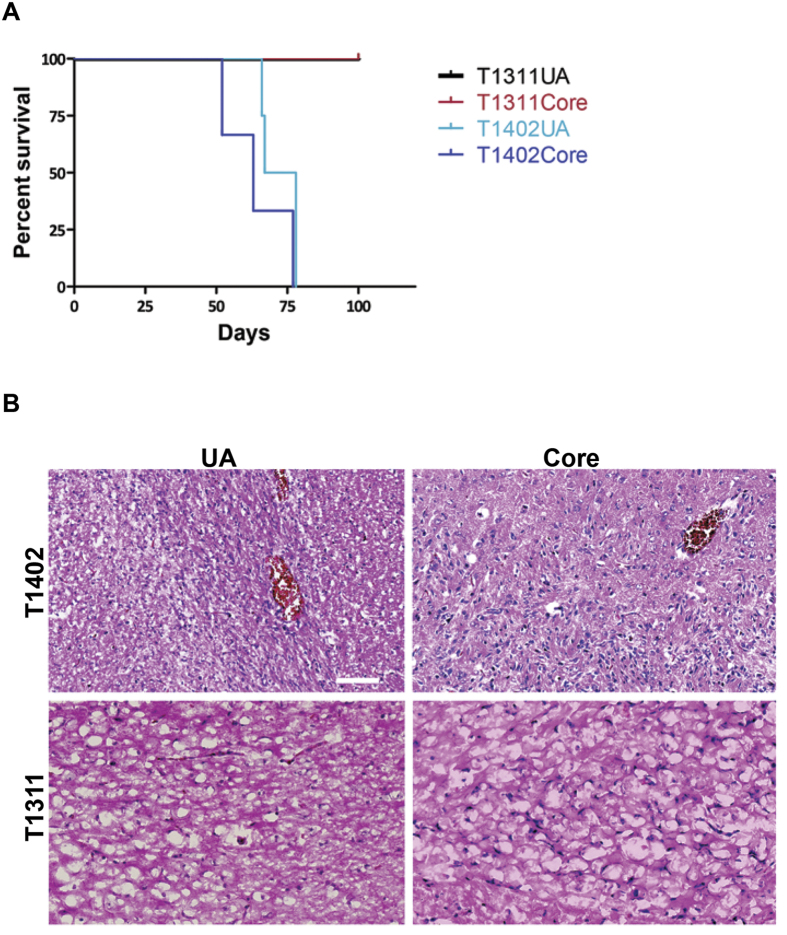
UA cells show similar tumorigenic potential to tumor core-derived cells. (A) Kaplan–Meier survival analysis of tumor cells derived from UA and core samples. There was no significant difference between the mice injected with T1402 UA (n = 4) and tumor core-derived cells (n = 3). All mice injected with T1402, Core or UA, showed neurological symptoms related to tumor growth within 11 weeks (P = 0.153, log-rank test). Mice injected with T1311, a secondary GBM, did not show any neurological symptoms and were sacrificed after 100 days. (**B**) Representative H&E staining of tumors induced by injecting cells from UA and tumor core that were grown under sphere conditions between P5-P8. Scale bar: 1000 μM.

To investigate the tumorigenic potential of UA-cells expanded in sphere conditions we used intracranial stereotactic injection of these cells and cells from tumor core biopsies into one hemisphere of immuno-compromised mice. Cells from UA and matched tumor core from two patients were used for transplantation experiments, each transplanted into 3–4 mice. Mice injected with cells from T1402 UA and core biopsy developed neurological symptoms and were sacrificed without significant difference in survival (P = 0.153, log-rank test). On the other hand, mice injected with cells from T1311 UA and core biopsy, a secondary GBM, did not show any neurological symptoms and were sacrificed after 100 days (Fig. 6A). Despite the absence of symptoms, cells from both types of samples were found to give rise to tumors (Fig. 6B).

### UA cells have a similar invasion pattern to tumor core cells *in vivo*

**Figure 7:**
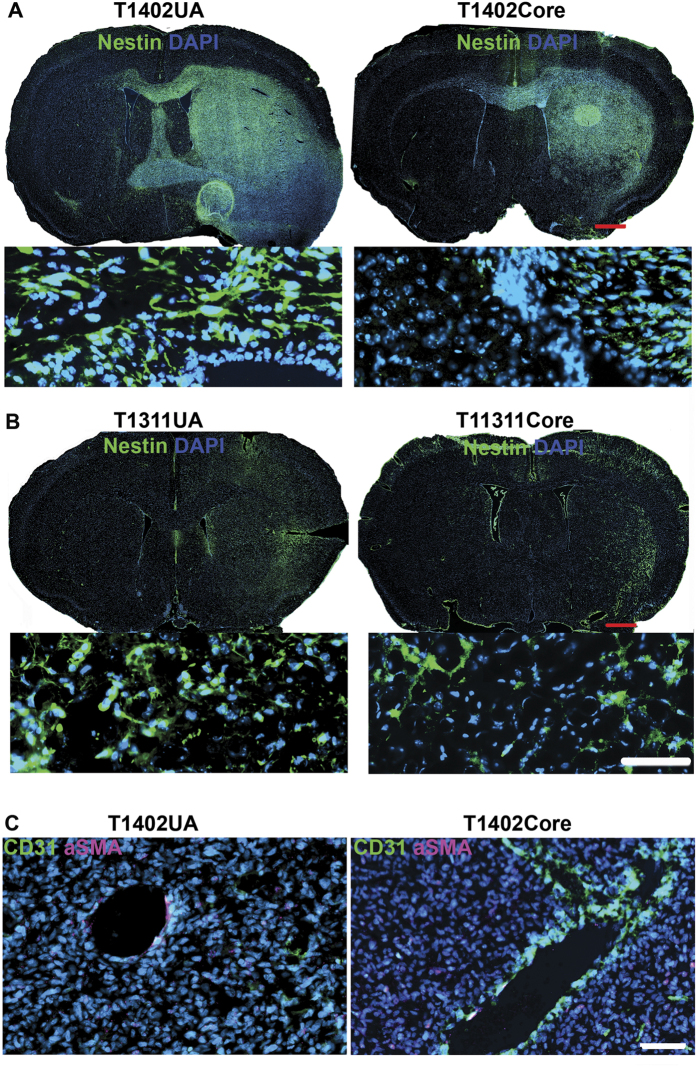
UA- and tumor core-derived cells have similar tumor invasion patterns *in vivo*. (**A**,**B**) A fluorescent overview image and zoom in field of a tumor induced by T1402 and T1311 UA- and tumor core-derived cells. Tissue sections were stained with human-specific Nestin (green) and DAPI. Both UA- and tumor core-derived cells of T1402 formed large tumors that infiltrated into the opposite hemisphere (**A**). While T1311 induced less invasive tumor cells that were dispersed within the same injection side. (**B**), Scale bar: 500 μM in overview images and 100 μM in zoom in. (**C**) A fluorescent image of tumors induced by UA- and tumor core-derived cells showing the angiogenesis in the tumor. Tissue sections were stained with CD31 (red), and αSMA (green) antibodies highlighting the blood vessels. Scale bar 100 μM.

Human-specific Nestin staining indicated that T1402UA and its corresponding core formed large invasive tumors that crossed into the opposite hemispheres (Fig. 7A), while T1311 gave rise to a small number of cells dispersed in hemisphere of the injection site (Fig. 7B). Angiogenesis was detected in T1402 UA- and core-induced tumors, but not those of T1311 (Figs 6B and 7C). Thus, cells from both UA and tumor core samples, expanded under sphere culture condition, showed similar tumorigenic potential. They both form highly invasive tumors or non-invasive tumors that mimic their parental tumor.

## Discussion

The increasing interest in cancer stem cell (CSC) biology and the use of these cells in protocols for immunological cancer therapy has put much focus on the development of cell culture techniques to expand CSCs. This applies also to the field of GBM research. The low cell yield and risk of necrotic tissue in tumor core biopsies has stimulated a search for other sources of GBM material. In addition, tumor core biopsies may not reveal the heterogeneity that is a characteristic of many GBMs. The intra-tumoral heterogeneity of GBM has been suggested to be one of the main reasons for the development of therapy resistance. For instance, intra-tumor heterogeneity of receptor tyrosine kinases EGFR and PDGFRA expression levels defines subpopulations with distinct growth factor response in GBMs^11^,^23^. To overcome the problem of intra-tumor heterogeneity, a multibiopsy sampling strategy combined with 5-aminolevulinic acid-based fluoresence guidance was suggested^11^. However, this strategy requires extra efforts in the operation theatre because it uses real-time sampling during patient operation. As well, the dissected tumor size by this technique is 2–3 mm^3^, which may create some difficulties during sample processing and with getting enough cells for culturing. Thus, there is an increasing need to use a cell source and culture strategies that do not suppress the features of intra-tumoral heterogeneity.

Our work suggests that UA samples could offer a way to retain tumor heterogeneity, as sonication and sucking of liquid and tissue fragments is a continuous process during a tumor operation, making the UA sample potentially the best candidate to represent global tumor heterogeneity. On a molecular level, the PCA analysis showed that there are paramount differences in the gene expression between the outlined groups of UA samples and Co samples. We identified 352 differentially expressed genes between UA and Core samples, in addition to several pathways that were differentially regulated and higher in UA samples, such as p53, notch and ECL interaction pathways. Also, several cancer related genes such as proto-oncogenes jun and fos were upregulated in UA samples. Furthermore, tumor subtyping showed that two patients have different subtype for UA samples and core biopsies. Also, targeted DNA sequencing showed that UA samples harbour many mutations that cannot be detected in tumor core biopsy. These data together indicate that UA samples cover the oncogenic molecular profile of GBM better than do regular tumor biopsies. Thus, this way of harvesting tumor tissue can represent some aspects of tumor heterogeneity that are missed in tumor biopsy.

Another important advantage of using UA samples is the superior number of viable cells. We showed that the viable cell number ranged between 33 × 10^6^–10 × 10^8^, bearing in mind that this was the cell yield of the processed part, and not of the total obtained UA samples. Viable cell yield can be a major problem in the use of tumor biopsies, where in two cases we failed to establish sphere culture from biopsies probably due to the low number of viable cells derived from the biopsies, while the matching UA samples gave good cell yield and successful sphere cultures.

We have shown that both UA and their matched tumor core biopsy samples express a range of stemness and proliferation markers, have multilineage neural differentiation potential, comparable tumorigenicity and tumor invasion pattern *in vivo*. The difference in the stemness and proliferation markers between matched UA and tumor core derived cultures could be an indicator of different tumor cell populations within the two samples. However, this did not affect the multilineage differentiation capability and tumorigencity of these cultures. Pellegatta and her colleagues (the 11^th^ Congress of the European Association of Neuro-Oncology, 2014) have reported that UA-derived cells have higher proliferation rates *in vitro* than tumor core-derived cells. Our data also showed that UA cells had a tendency towards higher proliferation rates *in vitro*, but this difference did not reach statistical significance. Others have previously reported the use of UA samples as archival tissue reserves for microarray studies and to improve histology analyses, which is usually dependent on tumor core biopsies only^16^,^17^. In one of their analyses they showed that microvascular proliferation was seen in a UA sample, but not in the matching core biopsy, which changed the diagnosis of the patient to high grade GBM^17^.

Recently, some studies have shown that there are live cells in UA samples that can be used for expanding cancer cells in cultures^18^,^19^. However, these studies have some methodological issues. One study compared 2 fractions, liquid and tissue fragments, of the same UA samples without comparison with the matched tumor core biopsy. In addition, it used a monolayer cell culture system with serum or serum free medium on a laminin coated surface, which is different from the classical sphere culture system used here^18^. The Jensen *et al.* study used UA samples to establish organotypic spheroid cultures which were maintained for 14 days^19^. Although organotypic spheroid culture has been suggested to preserve different tumor compartments of tumor stroma and cancer stem cells including their cellular connection, blood vessels and macrophages, they have disadvantages such as short life span in culture, (10–15 days), and they contain dead cells and undergo necrosis^19^,^24^,^25^. In our opinion, using UA samples in an organotypic spheroid culture system is unlikely to be a good option for clinical use, especially for vaccine therapy setup or drug target therapy. One important issue could be that UA samples contain a lot of normal brain cells, and 10–14 days is not enough time to deplete normal cells from the culture. Jensen *et al.* reported this observation in their work^19^. Thus, using these samples *ex vivo* to prime antigen presenting cells such as dendritic cells for subsequent activation of T cells might increase the risk of autoimmune diseases. Similarly, using this culture system for drug screening assays that depend on cell viability may give erroneous results due to the admixture of normal cells.

In our current work and previous work^22^ we have established sphere cultures from UA samples and used them for analysis after culture expansion for at least 40 days. To evaluate the risk of *in vitro* transformation, we performed high-resolution comparative genomic hybridization. The DNA analysis showed that the sphere cultures expressed chromosomal abnormalities related to the parental fresh tumor samples. Also, RNA quality control showed that the extracted RNA from these cultures is of high purity. However, the sphere culture system that we used has some known disadvantages. Most importantly, the success rate we have is only 75%. This is equal to or even higher than what has been previously reported^20^,^21^, but this means that it will not be possible to establish sphere cultures from more than 25% of the GBM samples. Thus, there is a need to optimize the sphere culture conditions or use another culture system to grow samples from this group of patients that do not respond to EGF and bFGF. Cell culture systems with enhanced surface attachment via laminin, or utilizing a culture protocol with a low serum component (1%FBS + TGFα + bFGF) may represent ways to establish successful cultures from these patient samples^26^,^27^. Furthermore, we noticed another important disadvantage for the sphere culture condition in the case of a secondary tumor that we used *in vivo*. Although the histology of the secondary GBM is largely indistinguishable from the primary GBM^28^, the transplanted sphere culture cells of this tumor gave an apparent low grade brain tumor in both the cases of UA and core biopsy. Interestingly, targeted NGS showed that cells enriched in sphere culture lost their EGFR and NF1 mutations that were expressed in the tumor core biopsy. These two genes are known to be GBM driver mutations and among the most frequently altered genes^29^. Recently it was shown that EGFR is one of the six mutations that characterize hypermutant samples induced by the GBM treatment protocol^30^.

In summary, we have performed a systematic study comparing UA samples and tumor core biopsy samples side-by-side. We show that UA samples, which are usually considered as biological waste, offer a significantly more abundant source for cells that can be expanded in culture under sphere conditions with a similar growth pattern to tumor core samples. We also show that UA cells express a range of CSC and proliferation markers with sample-specific differences from tumor core-derived cells. Both UA cells and tumor core-derived cells have multilineage neural differentiation potential, and they have similar tumorigenic potential and invasion patterns *in vivo*. Our molecular data indicate that UA samples cover tumor heterogeneity better than tumor core biopsies. These results, in addition to negative microbiology safety tests, and RNA quality control show that UA samples offer a huge and safe source for cells that can be expanded in culture and are qualified to be used in clinical vaccine therapies. However, to capture the essence of tumors’ cell heterogeneity, we believe that we need to combine both sources of samples, ie. The core biopsy and the UA sample.

## Methods

### Patient samples collection

UA samples from brain tumor surgery were acquired from 40 consenting patients, 33 GBM, 6 astrocytoma II and III, 2 ependymoma and 1 case of brain metastasis. Tumor core biopsies were obtained from 15 of those patients. Informed consents were obtained from all patients before getting the samples. All experimental procedures were carried out according to the guidelines of the Norwegian National Committee for Medical Research Ethics after being approved by the Regional Ethical Committee (REC South-East S-07321d). Tumors were histologically classified according to WHO criteria. The cells were obtained by ultrasonic aspiration with a Sonoca 300 ultrasonic dissector/aspirator (Söring, Quickborn, Germany) preoperatively.

### Sample processing and cell culture

Fresh tumor samples from both UA and tumor core biopsies were processed directly or within a few hours of the operation. 1 ml of the sample was grown in the BACTEC blood culture system to check for aerobic bacteria (BD Diagnostics, Shannon, Ireland). The tissue fragments in the liquid fraction were spun down, washed and then weighed. The collected tissue was divided into three fractions: one was snap frozen for protein and RNA extraction, one was cryopreserved in FBS/DMSO (BioChrome, VWR and Sigma), and one was processed into a single cell suspension. To obtain a single cell suspension UA tissue fragments were incubated with TrypLE™ Express (Gibco®, Life Technologies, Sweden) for 15 min at 37 °C. Pieces of tissue were further homogenized by pipetting and filtered through a 100 μm cell strainer (BD Falcon). The cell suspension was collected and washed with Leibowitz-15 (Invitrogen, Carlsbad, CA) and incubated with red blood cell lysis buffer, BD Pharm Lyse™ (BD Biosciences, Sweden) for 5 min at room temperature. Tumor core biopsy samples were minced into small pieces and then enzymaticly digested as were UA samples. Cells were washed again, counted and plated in serum free sphere culture conditions containing DMEM/F12-GlutaMAX medium (Life Technologies, Inc), 10 ng/ml bFGF, 20 ng/ml EGF (both R&D Inc, Minneapolis), 1:50 B27-supplement (Gibco®, Life Technologies), 100 U/ml Penicillin/Streptomycin of both (LonzaBioWhittaker), 1 ng/ml Heparin (Leo Pharma, Ballerup, Denmark), and HEPES 5 mM (Lonza, BioWhittaker). The cells were seeded at a density of 13000 cells/cm^2^ in non-treated cell culture flasks (Nunc, VWR). Cells were fed with EGF and bFGF twice a week and cell culture medium was refreshed on day 7 and cells were passaged every 10–14 days. Cell harvesting was done by enzymatic digestion, trypsin-EDTA (0.25%). Collected cells were washed, centrifuged, and resuspended in fresh medium or working solution buffer according to experimental plan. See supplementary method for details about the sample processing procedure. Only 24 GBM patient samples and 1 metastasis sample were able to be grown in sphere conditions, either as floating spheres or adherently. *In vitro* self-renewing was evaluated at early passage (P4-P6) previously through single cell sorting and serial limited dilution assay (1–3 cells/well) in a 96-well plate^22^. Also, we have tested this property at later passages (P9-P11) (Table S7). Good laboratory practice was followed and cell cultures were tested for mycoplasma occasionally.

### Flow cytometry analysis

Sphere culture cells from both UA samples and tumor core biopsies were used for fluorescence-activated cell sorting (FACS) analysis between passages P4-P9. Spheres were processed into single cells, washed with FACS buffer, PBS (Dulbecco’s Phosphate Buffered Saline, Lonza, BioWhittaker) containing 4% FBS and stained for 1 h at 4 °C. For intracellular staining, cells were first fixed and permeabilized using a *BD* Cytofix/Cytoperm™ Kit, and then incubated overnight with primary antibodies, after washing, cells were incubated with matched secondary antibodies for 2 h. Directly conjugated or primary and secondary antibodies are shown in supplementary information (Table S8). Cells were then washed and analyzed by a LSRFortessa™ cell analyzer (BD Bioscience). At least 10,000 events were counted. The Fluorescence Minus One Control (FMOs) and secondary antibody staining (without primary antibody) were used as negative controls to set the gate for each marker. FlowJo software was used for data analysis.

### Neural differentiation

Single cells dissociated from sphere cultures were plated on cover slips (Thermo Scientific) in 24 well plates supplied with differentiation medium. This medium consisted of DMEM/F12-GlutaMAX medium supplemented with 4% FBS, pencillin/streptomycin, HEPES, B27-supplemented with retinoic acid (Invitrogen). The cover slips were coated with poly-L-ornithine (28 μg/cm^2^, 0.1 mg/mL; Sigma-Aldrich) plus laminin for 48 h at 37 °C. Before plating, coated cover slips were washed three times in sterile H_2_O. The cells were differentiated for two weeks and medium was changed every 3–4 days. Undifferentiated cells were used as a control.

### Immunocytochemistry and immunohistochemistry

For immunofluorescent cell labelling, cells were grown on cover slips incubated with the indicated primary antibodies overnight, washed 2 × 2 minutes in PBS followed by 1 × 2 minutes in TBST and subsequently incubated with appropriate secondary antibodies for 2 h at room temperature, gently washed 2 × 2 min in PBS, then stained for 10 minutes in Hoechst (1:1000, Sigma Aldrich) and mounted with Prolong Gold anti-fade reagent (Invitrogen).

For dissected mouse brain tissue, the brain was fixed in 4% paraformaldehyde for 24 hours, and cryoprotected in 20% sucrose for another 24 hours, then snap frozen using liquid nitrogen. 14 micrometer cryostat sections were prepared and kept at −20 °C. For immunohistochemistry staining, frozen sections were allowed to be defrosted for 15 minutes, rehydrated 3 × 5 minutes in PBS, and then 2 × 5 minutes in Tris-Buffered Saline Tween-20 (TBST) and incubated with blocking buffer (5% milk powder in TBST and 10% normal serum of secondary antibody host) for 30 minutes at room temperature. The primary antibodies, human-specific Nestin, CD31 and alpha smooth muscle actin (αSMA) from Abcam were used for this staining. Slides were incubated overnight with primary antibodies at 4 °C, washed 2 × 5 minutes in PBS followed by 2 × 5 minutes in TBST, and then incubated with secondary antibodies conjugated to Alexa Fluor 488, Alexa Fluor 594, Alexa Fluor 647 (1:500), incubated overnight at 4 °C, washed 3 × 5 minutes in PBS and stained for 10 minutes in Hoechst (1:1000) before being mounted with Prolong Gold anti-fade reagent and kept to dry for a while before microscopy.

### Microscopy

For confocal microscopy, cover slips were examined at 40x magnification using a Zeiss Meta 510 confocal laser-scanning microscope. The same laser intensity and confocal settings were used for each comparison. Image processing and analysis were carried out with Zen lite 2012 (Carl Zeiss, Jena, Germany) and Adobe Photoshop (Adobe Systems).

High-resolution images of histological sections were acquired using an automated slide scanner system, and image analysis was performed by the Axio Scan Z1 from Carl Zeiss. (Axio Scan Z1, Carl Zeiss MicroImaging, Jena, Germany).

### *In Vivo* transplantation

The experimental protocol was approved by the Norwegian National Animal Research Authority project licence no FOTS-id 4785 and 5940. The animal experiments were performed in accordance with the European Convention for the Protection of Vertebrate Animals used for Experimental and Other Scientific Purposes (ETS 123) and The Guide for the Care and Use of Laboratory Animals, 8th edition (NRC 2011, National Academic Press). Six to eight-weeks old severe combined immunodeficiency (SCID) mice were purchased from Scanbur (Scanbur AS, Denmark). Sphere cultures were processed into single cells and 100 000 tumor cells in 4 μl neurobasal medium were *stereotactically* injected as previously described^31^. Mice were allowed to recover after operations; their health status was closely monitored, and they were immediately euthanized when they started showing neurological symptoms.

### DNA extraction and targeted next generation sequencing (NGS)

Total genomic DNA was extracted from tissue and sphere culture derived cells using the Blood & Cell Culture DNA Mini Kit (Qiagen, Hilden, Germany) following the handbook protocol (Tissue Protocol, 02/2003). NGS libraries were generated with the Ion AmpliSeq™ Library Preparation Kit (Life Technologies) and our customized gene panel using ten nanograms of genomic DNA per primer pool for library preparation. Our targeted gene panel includes 29 genes, 18 genes relevant to the characterization of human gliomas, 6 genes frequently mutated in pediatric gliomas and 5 genes mutated in neurofibromatoses or schwannomatosis (Table S4). Amplification and adapter ligation were performed according to the manufacturer’s protocol (MAN0006943 Rev.4.0, Life Techologies). The libraries were bar coded during the ligation reaction using the Ion Xpress™ Barcode Adapter Kit (Life Technologies). Emulsion-PCR was performed on the Ion OneTouch™ 2 System (Life Technologies) using the Ion PGM™ Hi-Q View OT2 Kit (MAN0014580 Rev.A.0. Life Technologies). Afterwards, template-positive Ion Sphere Particles (ISPs) were enriched on the Ion OneTouchTM ES instrument (Life Technologies) according to the manufacturer’s recommendations. Sequencing runs were performed on the Ion Torrent Personal Genome Machine (PGM) ™. For Ion Torrent PGM™ sequencing, we used Ion 318™ chips and the Ion PGM™ HI-Q Sequencing Kit v2 (MAN0010863 Rev.B.0, Life Technologies). Amplicon sequences were aligned to the human reference genome GRCh37 (hg19) in the target region of our custom gene panel using Torrent Suite™ software 5.0. SNVs calling was performed using the Variant Caller (v5.0.4.0) on the Ion Torrent Browser and the predefined parameter set ‘Generic - PGM - Somatic - Low Stringency’. Variants were filtered for the following parameters: UCSC Common SNPs = Not In; dbSNP = Not IndbSNP = Not In; Location in utr_3, nonCoding, upstream, exonic_nc, exonic, intergenic, downstream, ncRNA, utr_5, unknown, splicesite_5, splicesite_3; Filtered Coverage > = 100.

### RNA isolation

Total RNA was extracted using miRNeasy Mini Kit (Qiagen, Hilden, Germany). Around 2–5 × 10^6^ of fresh cells or 1.5–2 × 10^6^ cultured cells were incubated in 0.7 ml Qiazol (Qiagen Sciences, Maryland, USA) for 5 min at RT, then 70 μl 1-Bromo-3-chloropropane (Sigma-Adrich, St.Louis, USA) was added and vigorously shaken and centrifuged at 12000 g for 10 min at 4 °C. After this, the acqueous phase was collected and transferred to a new tube containing 0.5 ml of 100% Ethanol and mixed thoroughly. The mixture was pipetted onto an RNeasy Mini column and centrifuged (12000 g, 15 sec, RT) after which the flow-through was discarded. Then 0.5 ml RPE buffer was added to the column and centrifuged (12000 g, 15 sec, RT). This step was repeated again for 1 min. 25 ul of RNase-free water was used to elute the RNA. Purity and quantity were measured by a Nanodrop spectrophotometer. Also, RNA concentration and RNA quality control were determined using a 2100 Bioanalyzer. Only RNA that had a RIN value higher than four was used.

### Statistical and bioinformatic analysis

Survival analysis and the Mantel-Cox log-rank test were performed using GraphPad Prism 6 (GraphPad Software, LA Jolla, CA). A p value less than 0.05 was considered significant. The quantification of the markers in FACS analysis is shown as a percentage of the mean ± SEM. A non-parametric t-test and Mann-Whitney test were used to check the significance value.

RNA was amplified and hybridized to Illumina HumanHT-12 V4.0 expression beadchip (platform GPL10558). Microarray data were imported to the Microarray data analysis tool J-Express (Molmine, Bergen, Norway)^32^,^33^ and quantile normalized. For hierarchical clustering (HCL) the data were log_2_ transformed and normalized to the mean for graphical presentation. For hierarchical clustering^34^ the average distance was calculated using UPGMA (unweighted pair group method with arithmetic mean) while we used Pearson correlation as a distance measure. Differentially expressed genes (DEGs) were identified using the grouping function and fold change analysis in J-Express. Briefly, the microarray data were quantile normalized and filtered using standard deviation. The 7715 most variable genes were selected for further analysis while the samples were grouped into two groups. The two groups consisted of a) Fresh tissue-core samples and b) Fresh tissue Ultrasound aspirates (UA). Using the cut-off fold change value of >2, 352 DEGs were selected. The HCL chart of these genes is shown in Supplemental (Figure S8). By setting the cut-off value to >1.5 we identified 1176 DEGs that were submitted to the DAVID web-based functional annotation tool suites v 6.7 (https://david.ncifcrf.gov)^35^,^36^. Of these 937 genes were recognized by DAVID and further analyzed for pathways.

For principal component analysis (PCA)^37^, the quantile normalized data were exported from J-Express as tabular data and imported to the MultiExperiment Viewer MeV v4.8.1 (http://www.tm4.org/mev.html) for visualization. Microarray data comply with MIAME standards and have been submitted to the Gene Expression Omnibus^38^ with GEO accession number (GSE84707). Subtyping of GBM tissues and cell lines was done according to a12-gene signature^22^. The expression data for 178 GBMs from TCGA^39^ were downloaded from https://tcga-data.nci.nih.gov/docs/publications/gbm_exp/ and processed using J-Express.

## Additional Information

**Accession Code**: Microarray data have been deposited in the Gene Expression Omnibus (GEO) database (accession number GSE84707)

**How to cite this article**: Behnan, J. *et al.* Ultrasonic Surgical Aspirate is a Reliable Source For Culturing Glioblastoma Stem Cells. *Sci. Rep.* **6**, 32788; doi: 10.1038/srep32788 (2016).

## Supplementary Material

Supplementary Information

Supplementary Table S3

Supplementary Table S5

Supplementary Table S6

## Figures and Tables

**Table 1:** Cell yield obtained from ultrasonic aspiration samples compared to tumor core biopsies.

Sample	Total weight (g)	Processed (g)	Total cell count	Total viable cells	Viability	Yield (viable cells/g)
T1311UA	14.4	8.8	443 × 10^6^	293 × 10^6^	66%	24 × 10^6^
T1311Core			3.1 × 10^6^	2.8 × 10^6^	90%	
T1341UA	47	11	862 × 10^6^	338 × 10^6^	39%	30 × 10^6^
T1341Core			25 × 10^6^	8.3× 10^6^	33%	
T1344UA	33	11	538 × 10^6^	352 × 10^6^	65%	32 × 10^6^
T1344Core	(necrotic)		510 × 10^6^	110 × 10^6^	22%	
T1348UA	20	11.5	1239 × 10^6^	494 × 10^6^	40%	43 × 10^6^
T1348Core			113 × 10^6^	42 × 10^6^	37%	
T1349UA	18	8.5	505 × 10^6^	306 × 10^6^	61%	36 × 10^6^
T1349Core			39.6 × 10^6^	15.6 × 10^6^	39%	
T1370UA	21	9	617 × 10^6^	407 × 10^6^	88%*	45 × 10^6^
T1370Core			11.4 × 10^6^	9 × 10^6^	79%*	
T1402UA	20	12	204 × 10^6^	150 × 10^6^	82%*	13 × 10^6^
T1402Core			1.5 × 10^6^	9 × 10^5^	60%	
T1406UA	13	5	53 × 10^6^	33 × 10^6^	68%*	7 × 10^6^
T1406Core			3.6 × 10^5^	2.5 × 10^5^	69%*	
T1407UA	10.5	5.5	180 × 10^6^	88 × 10^6^	71%*	16 × 10^6^
T1407Core			21 × 10^6^	10 × 10^6^	83%*	
T1408UA	12	5	209 × 10^6^	119 × 10^6^	89%*	23.4 × 10^6^
T1408Core			3.1 × 10^6^	2.1 × 10^6^	77%*	
T1412UA	77	15	403 × 10^6^	286 × 10^6^	81%*	19 × 10^6^
T1412Core	15		338 × 10^6^	57 × 10^6^	17%*	3.8 × 10^6^
T1413UA	20	10	627 × 10^6^	380 × 10^6^	79%*	38 × 10^6^
T1413Core	Small biopsy		6 × 10^5^	3.7 × 10^5^	61%*	
T1416UA	9.5	4.7	97.5 × 10^6^	62.5 × 10^6^	85%*	13.3 × 10^6^
T1416Core			12.6 × 10^6^	11.6 × 10^6^	89%*	
T1417UA	9.9	5	62.5 × 10^6^	32.5 × 10^6^	64%*	6.5 × 10^6^
T1417Core	Small biopsy		2.6 × 10^5^	1.4 × 10^5^	55%*	
T1422UA	13	6.7	300 × 10^6^	222 × 10^6^	74%*	33 × 10^6^
T1422Core			8 × 10^6^	6 × 10^6^	80%*	
T1435UA	21	11	1508 × 10^6^	1040 × 10^6^	70%*	95 × 10^6^
T1435Core			26 × 10^6^	19 × 10^6^	72%*	
T1437UA	13	9.8	209 × 10^6^	158 × 10^6^	75%*	16 × 10^6^
T1437Core			97 × 10^6^	57 × 10^6^	66%*	
Mean±SEM (UA)			473.9 ± 24.1 × 10^6^	280.1 ± 14.2 × 10^6^	70.4 ± 0.9%	29 ± 21 × 10^6^
Mean±SEM (Core)			71.2 ± 8.2 × 10^6^	20.7 ± 1.8 × 10^6^	60.5 ± 1.4%	
P-value			0.0005(***)	0.0001(***)	0.1(NS)	

Abbreviations: (g), gram; after RBC, *indicates viability after red blood lysis buffer treatment. All data are Mean ± SEM, Mann-Whitney U test. (***P ≤ 0.001); NS, Not significant.
