# Supplementary material for: Ultrasonic Surgical Aspirate is a Reliable Source For Culturing Glioblastoma Stem Cells
Source: Sci Rep. 2016 Sep 8;6:32788. doi: 10.1038/srep32788 (PMC5015049; doi:10.1038/srep32788)
Supplement: Supplementary Information [file srep32788-s1.pdf]

Supplementary information

Ultrasonic Surgical Aspirate is a Reliable Source For Culturing Glioblastoma Stem Cells

Jinan Behnan, Biljana Stangeland,Tiziana.Langella, Gaetano Finocchiaro, Wayne Murrell and Jan E. Brinchmann

Inventory of Supplemental information

Supplemental data

Figure S1 related to Figure 2

Figure S2 related to Figure 2

Figure S3 related to Figure 2

Figure S4 related to Figure 2

Figure S5 related to Figure 2

Figure S6 related to Figure 2

Figure S7 related to Figure 2

Figure S8 related to Figure 2

Supplementary Figure legends

Table S1 related to Figure 1

Table S2 related to Figure 1

Table S3 related to Figure 2 (spreadsheet excel file, The excel file contains the list of the 1176 differentially expressed genes (DEGs), gene ontology according to DAVID database (see methods for details) and the list of pathways and the genes differentially regulated between the groups Fr-Co and Fr-UA**).**

Table S4 related to Figure 3

Table S5 related to Figure 3 (spreadsheet excel file)

Table S6 related to Figure 4 (spreadsheet excel file)

Table S7 related to Methods

Table S8 related to Methods

Supplementary Method (Protocol of Preparing single cell suspension from ultrasonic aspiration of brain tumor sample).

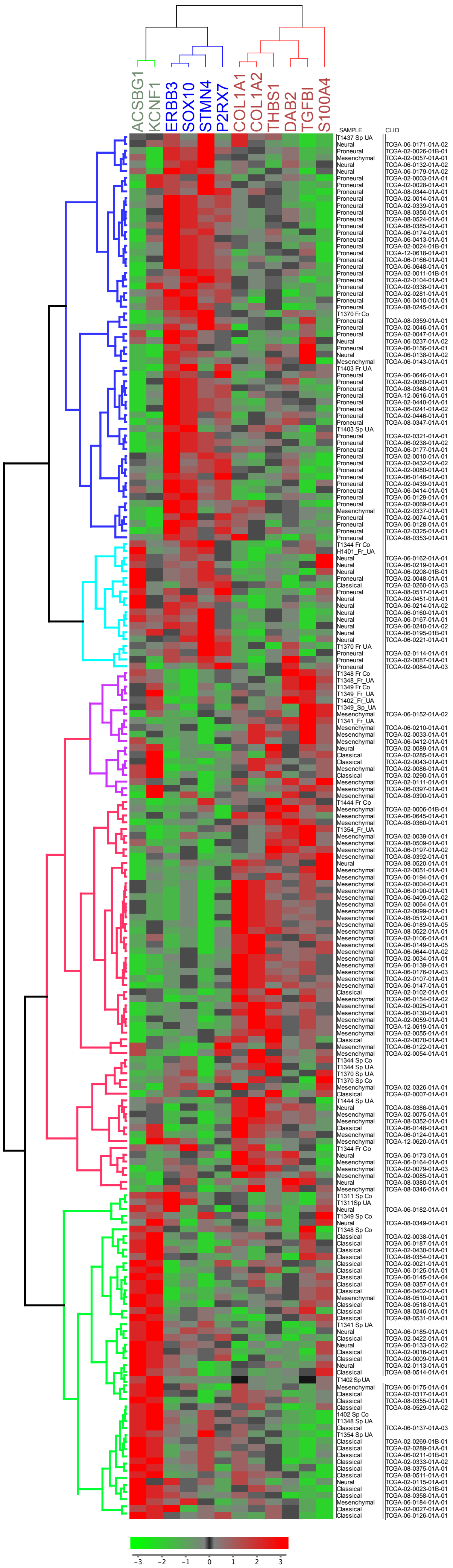

Distance metrics: Pearson Correlation
Linkage: COMPLETE

Figure S2

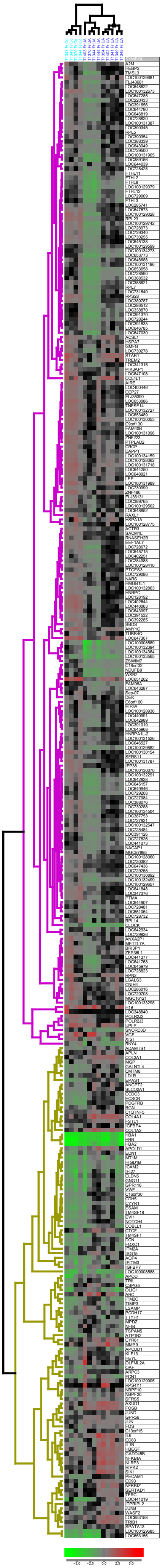

Distance metrics: Pearson Correlation
Linkage: WPGMA

Figure S3

Pathway:Notch signaling pathway

Pathway information generated by KEGG. ☑ **Stop Blinking**

Help

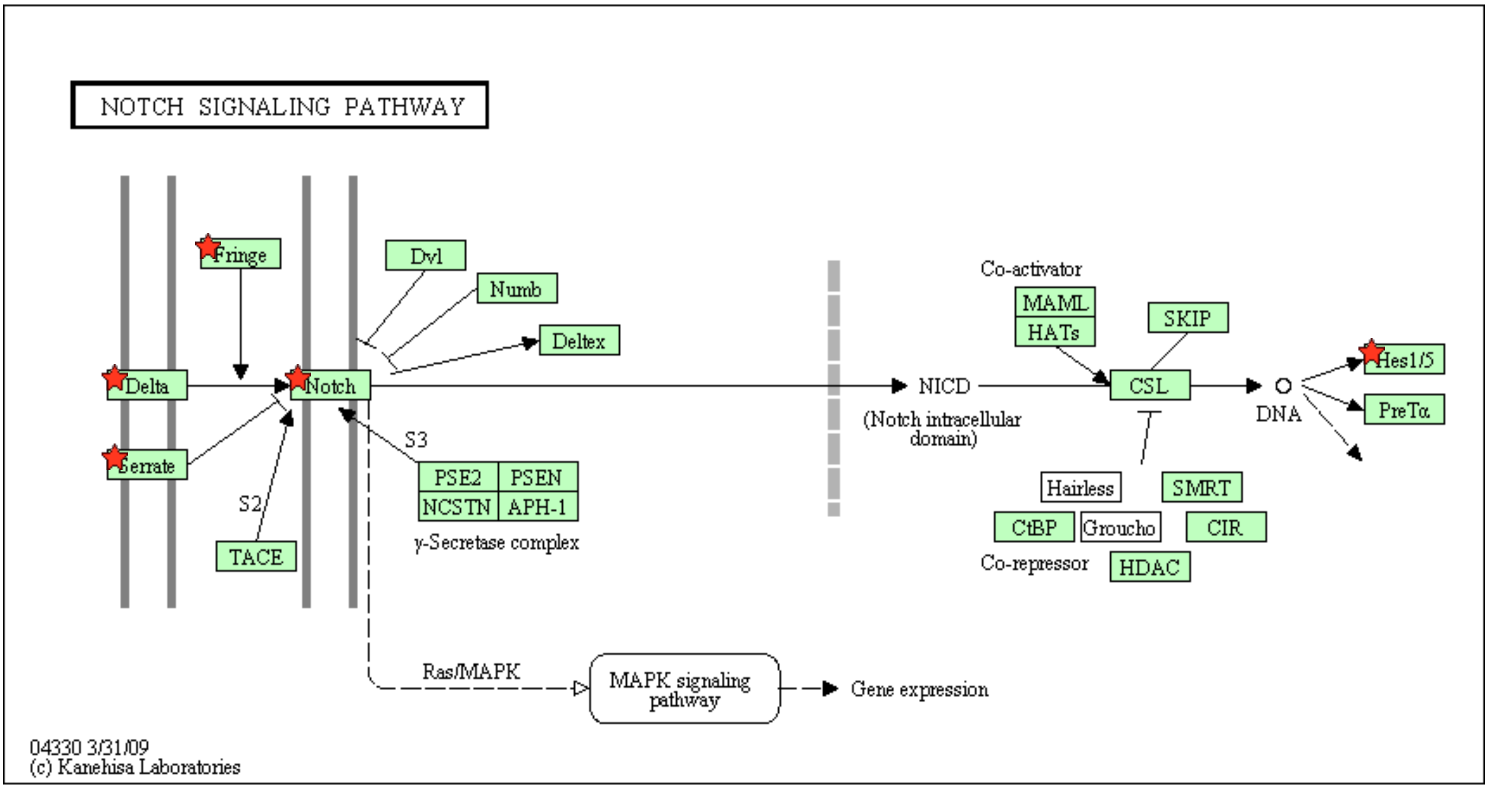

List genes are shown in red

DAVID Gene Name
ADAM metallopeptidase domain 17
C-terminal binding protein 1
C-terminal binding protein 2
CREB binding protein
E1A binding protein p300
K(lysine) acetyltransferase 2A
K(lysine) acetyltransferase 2B
LFNG O-fucosylpeptide 3-beta-N-acetylglucosaminyltransferase
MFNG O-fucosylpeptide 3-beta-N-acetylglucosaminyltransferase
Notch homolog 1, translocation-associated (Drosophila)
Notch homolog 2 (Drosophila)
Notch homolog 3 (Drosophila)
Notch homolog 4 (Drosophila)
RFNG O-fucosylpeptide 3-beta-N-acetylglucosaminyltransferase
SNW domain containing 1
anterior pharynx defective 1 homolog A (C. elegans)
corepressor interacting with RBPJ
delta-like 1 (Drosophila)
delta-like 3 (Drosophila)
delta-like 4 (Drosophila)
deltex 3-like (Drosophila)
deltex homolog 1 (Drosophila)
deltex homolog 2 (Drosophila)
deltex homolog 3 (Drosophila)
deltex homolog 4 (Drosophila)
dishevelled, dsh homolog 1 (Drosophila); dishevelled, dsh homolog 1 (Drosophila)-like 1
dishevelled, dsh homolog 2 (Drosophila)
dishevelled, dsh homolog 3 (Drosophila)
hairy and enhancer of split 1, (Drosophila)
hairy and enhancer of split 5 (Drosophila)
histone deacetylase 1
histone deacetylase 2
jagged 1 (Alagille syndrome)
jagged 2
mastermind-like 1 (Drosophila)
mastermind-like 2 (Drosophila)
mastermind-like 3 (Drosophila)

nicastrin
nuclear receptor co-repressor 2
numb homolog (Drosophila)
numb homolog (Drosophila)-like
pre T-cell antigen receptor alpha
presenilin 1
presenilin 2 (Alzheimer disease 4)
presenilin enhancer 2 homolog (C. elegans)
recombination signal binding protein for immunoglobulin kappa J region
recombination signal binding protein for immunoglobulin kappa J region-like

Show All List Genes

Show All Pathway Genes

Figure S4

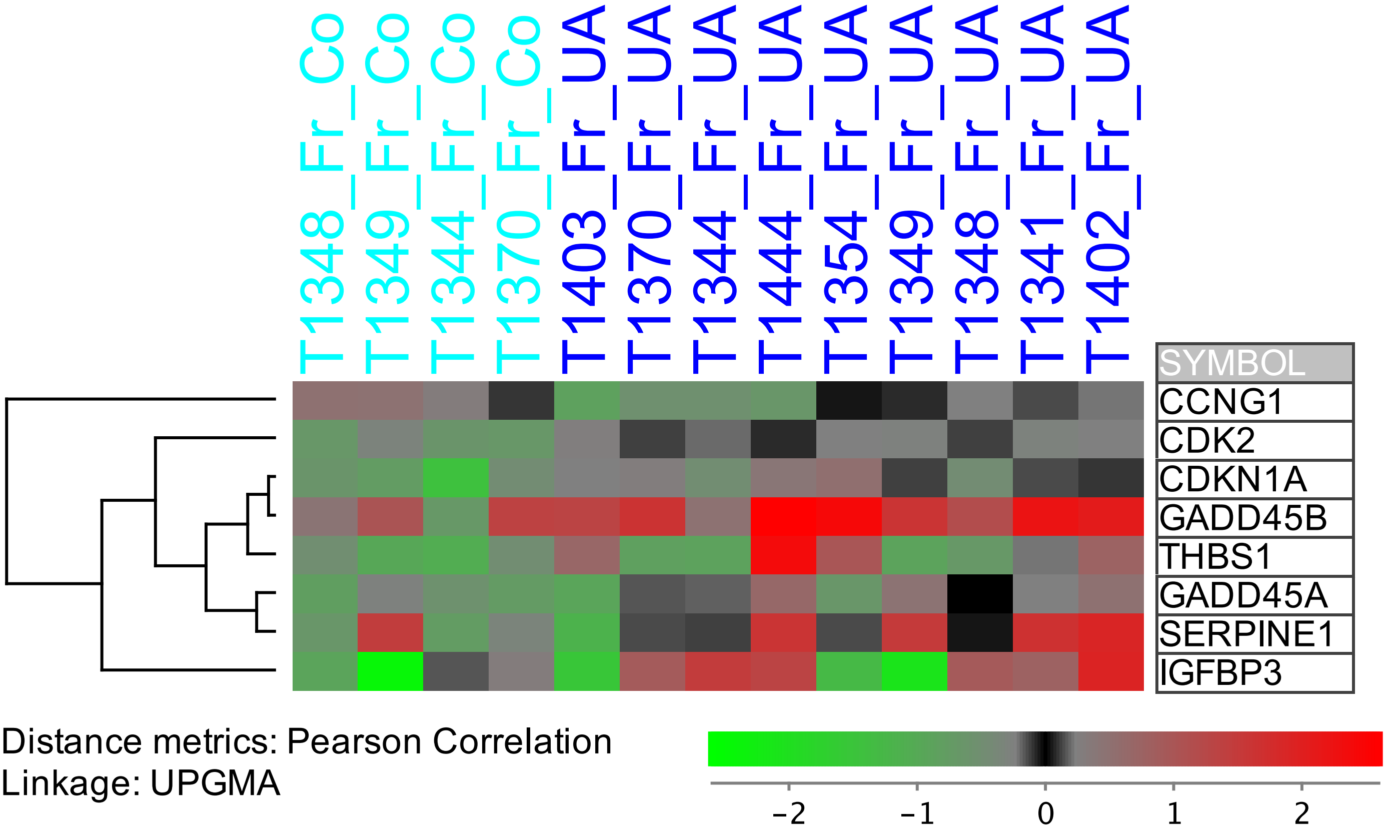
T1348_Fr_Co
T1349_Fr_Co
T1344_Fr_Co
T1370_Fr_Co
T1403_Fr_UA
T1370_Fr_UA
T1344_Fr_UA
T1444_Fr_UA
T1354_Fr_UA
T1349_Fr_UA
T1348_Fr_UA
T1341_Fr_UA
T1402_Fr_UA
SYMBOL
CCNG1
CDK2
CDKN1A
GADD45B
THBS1
GADD45A
SERPINE1
IGFBP3
Distance metrics: Pearson Correlation
Linkage: UPGMA
−2
−1
0
1
2

Pathway:p53 signaling pathway

Pathway information generated by KEGG. Stop Blinking

Help

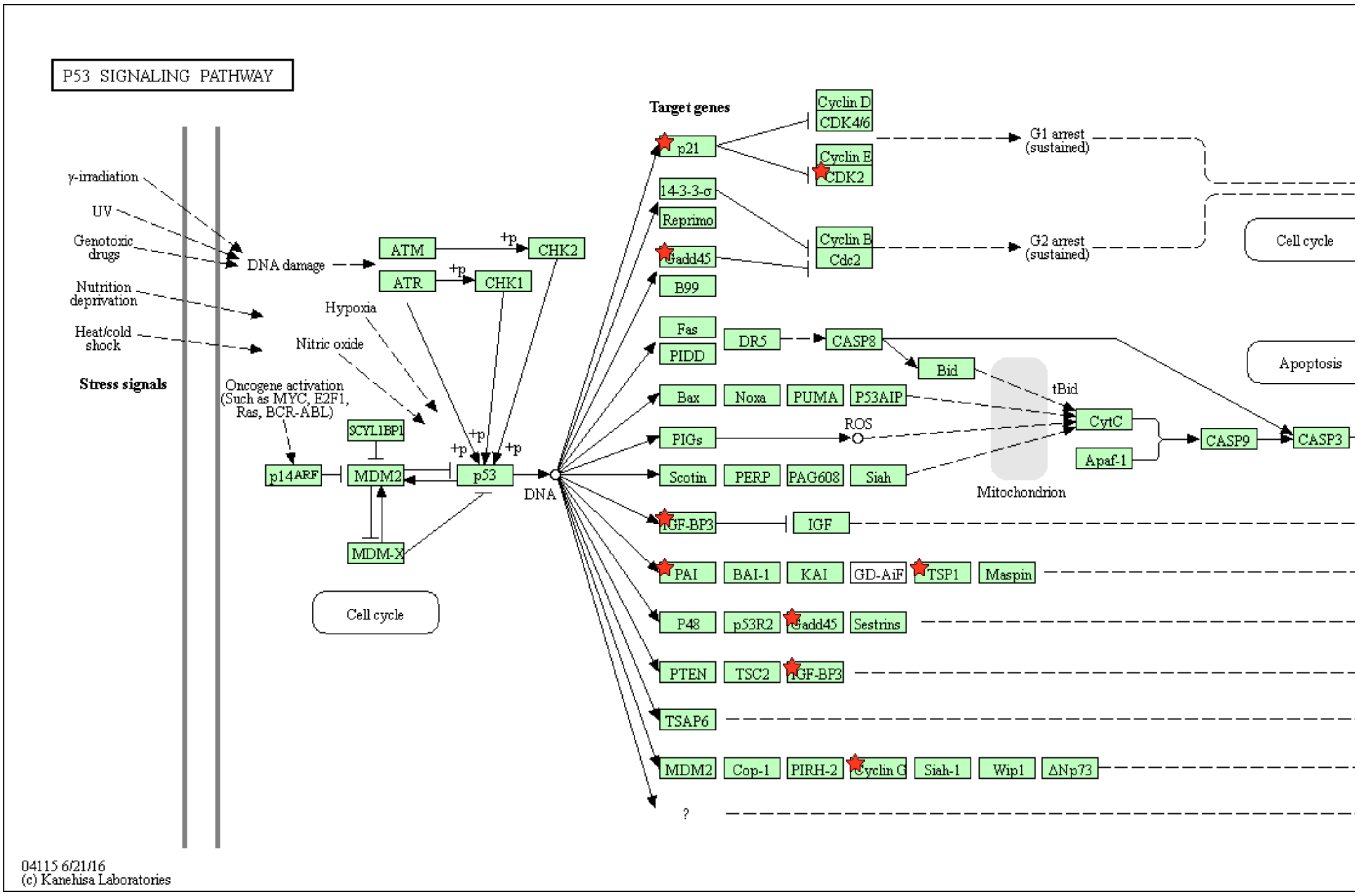

List genes are shown in red

DAVID Gene Name
BCL2 binding component 3
BCL2-associated X protein
BH3 interacting domain death agonist
CD82 molecule
CHK1 checkpoint homolog (S. pombe)
Fas (TNF receptor superfamily, member 6)
G-2 and S-phase expressed 1
Mdm2 p53 binding protein homolog (mouse)
Mdm4 p53 binding protein homolog (mouse)
PERP, TP53 apoptosis effector
STEAP family member 3
apoptotic peptidase activating factor 1
ataxia telangiectasia and Rad3 related; similar to ataxia telangiectasia and Rad3 related protein
brain-specific angiogenesis inhibitor 1
caspase 3, apoptosis-related cysteine peptidase
caspase 8, apoptosis-related cysteine peptidase
caspase 9, apoptosis-related cysteine peptidase
cell division cycle 2, G1 to S and G2 to M
cyclin B1
cyclin B2
cyclin B3
cyclin D1
cyclin D2
cyclin D3
cyclin E1
cyclin E2
cyclin G1
cyclin G2
cyclin-dependent kinase 2
cyclin-dependent kinase 4
cyclin-dependent kinase 6
cyclin-dependent kinase inhibitor 1A (p21, Cip1)
cyclin-dependent kinase inhibitor 2A (melanoma, p16, inhibits CDK4)
cytochrome c, somatic
damage-specific DNA binding protein 2, 48kDa
etoposide induced 2.4 mRNA
growth arrest and DNA-damage-inducible, alpha
growth arrest and DNA-damage-inducible, beta
growth arrest and DNA-damage-inducible, gamma
insulin-like growth factor 1 (somatomedin C)
insulin-like growth factor binding protein 3

leucine-rich repeats and death domain containing
phorbol-12-myristate-13-acetate-induced protein 1
phosphatase and tensin homolog; phosphatase and tensin homolog pseudogene 1
protein kinase CHK2-like; CHK2 checkpoint homolog (S. pombe); similar to hCG1983233
protein phosphatase 1D magnesium-dependent, delta isoform
reprimo, TP53 dependent G2 arrest mediator candidate
ribonucleotide reductase M2 B (TP53 inducible)
ribonucleotide reductase M2 polypeptide
ring finger and CHY zinc finger domain containing 1
ring finger and WD repeat domain 2
serpin peptidase inhibitor, clade B (ovalbumin), member 5
serpin peptidase inhibitor, clade E (nexin, plasminogen activator inhibitor type 1), member 1
sestrin 1
sestrin 2
sestrin 3
seven in absentia homolog 1 (Drosophila)
shisa homolog 5 (Xenopus laevis)
similar to Serine-protein kinase ATM (Ataxia telangiectasia mutated) (A-T, mutated); ataxia telangiectasia mutated
stratifin
thrombospondin 1
tuberous sclerosis 2
tumor necrosis factor receptor superfamily, member 10b
tumor protein p53 inducible protein 3
tumor protein p53 regulated apoptosis inducing protein 1
tumor protein p53
tumor protein p73
zinc finger, matrin type 3

Show All List Genes

Show All Pathway Genes

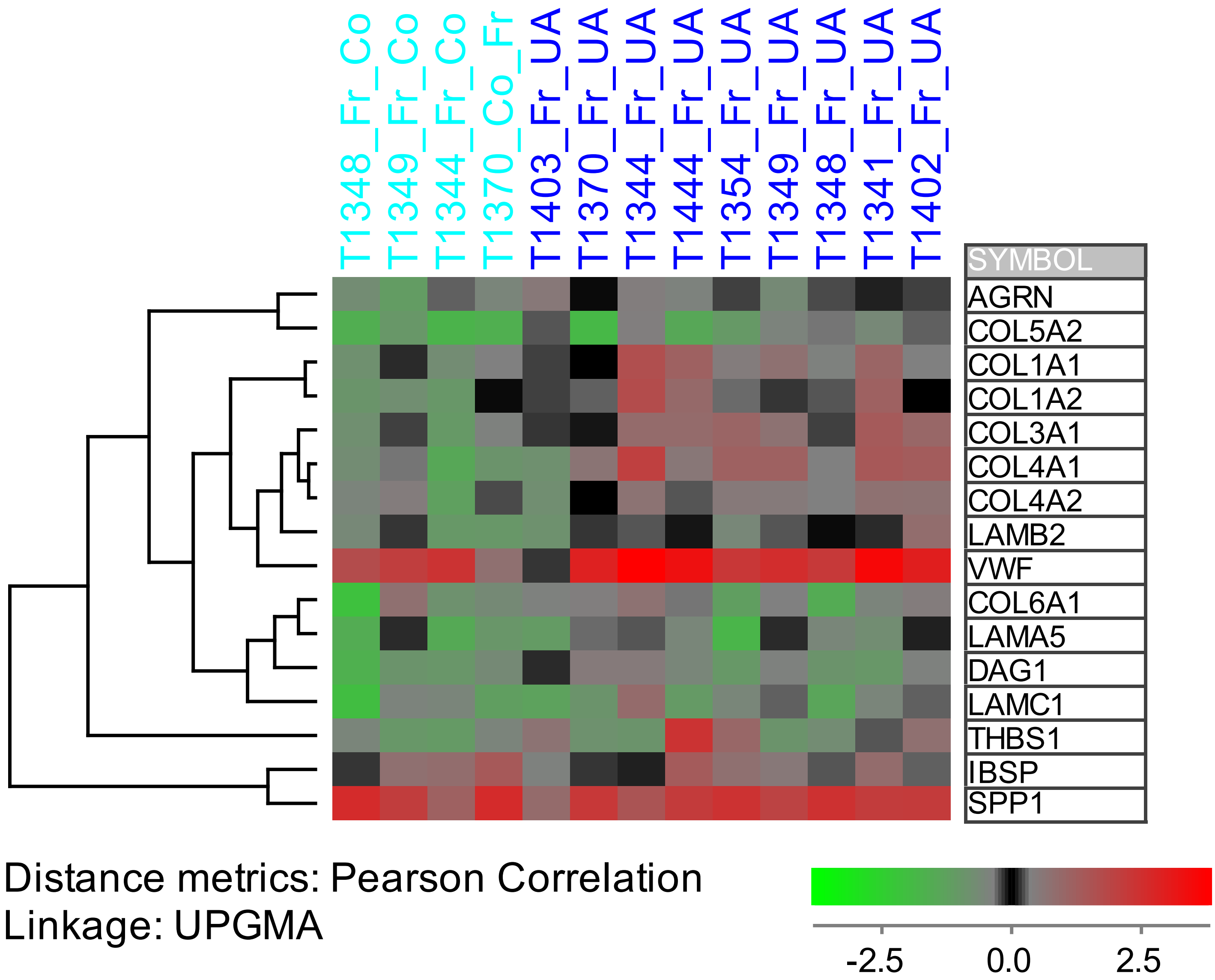
T1348_Fr_Co
T1349_Fr_Co
T1344_Fr_Co
T1370_Co_Fr
T1403_Fr_UA
T1370_Fr_UA
T1344_Fr_UA
T1444_Fr_UA
T1354_Fr_UA
T1349_Fr_UA
T1348_Fr_UA
T1341_Fr_UA
T1402_Fr_UA
SYMBOL
AGRN
COL5A2
COL1A1
COL1A2
COL3A1
COL4A1
COL4A2
LAMB2
VWF
COL6A1
LAMA5
DAG1
LAMC1
THBS1
IBSP
SPP1
Distance metrics: Pearson Correlation
Linkage: UPGMA
-2.5
0.0
2.5

Figure S7

Pathway:ECM-receptor interaction

Pathway information generated by KEGG. ☑ **Stop Blinking**

Help

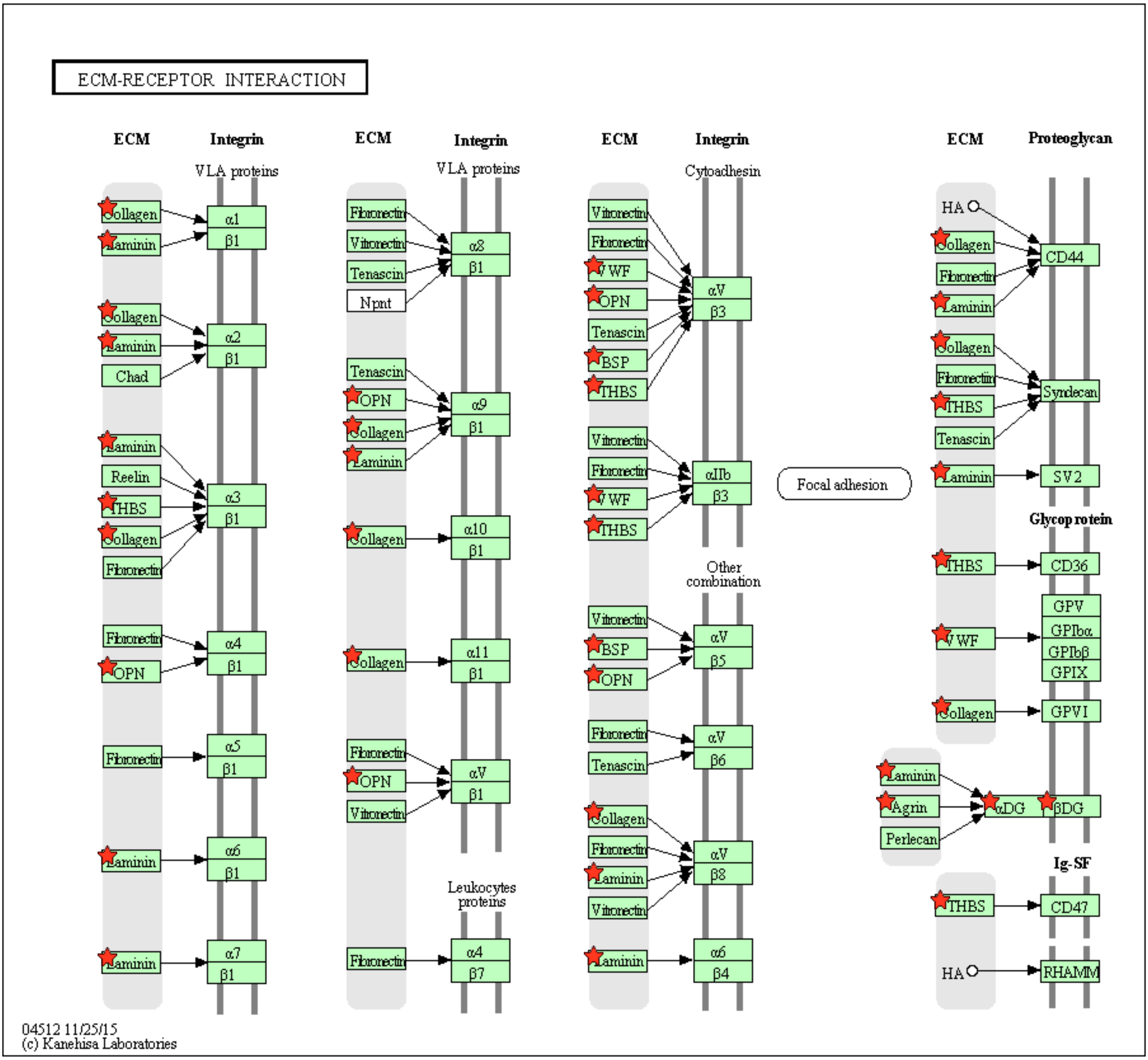

List genes are shown in red

DAVID Gene Name
CD36 molecule (thrombospondin receptor)
CD44 molecule (Indian blood group)
CD47 molecule
agrin
cartilage oligomeric matrix protein
chondroadherin
collagen type VI alpha 6
collagen, type I, alpha 1
collagen, type I, alpha 2
collagen, type II, alpha 1
collagen, type III, alpha 1
collagen, type IV, alpha 1
collagen, type IV, alpha 2
collagen, type IV, alpha 4
collagen, type IV, alpha 6
collagen, type V, alpha 1
collagen, type V, alpha 2

collagen, type V, alpha 3
collagen, type VI, alpha 1
collagen, type VI, alpha 2
collagen, type VI, alpha 3
collagen, type XI, alpha 1
collagen, type XI, alpha 2
dystroglycan 1 (dystrophin-associated glycoprotein 1)
fibronectin 1
glycoprotein IX (platelet)
glycoprotein Ib (platelet), alpha polypeptide
glycoprotein Ib (platelet), beta polypeptide
glycoprotein V (platelet)
glycoprotein VI (platelet)
heparan sulfate proteoglycan 2
hyaluronan-mediated motility receptor (RHAMM)
integrin, alpha 1
integrin, alpha 10
integrin, alpha 11
integrin, alpha 2 (CD49B, alpha 2 subunit of VLA-2 receptor)
integrin, alpha 2b (platelet glycoprotein IIb of IIb/IIIa complex, antigen CD41)
integrin, alpha 3 (antigen CD49C, alpha 3 subunit of VLA-3 receptor)
integrin, alpha 4 (antigen CD49D, alpha 4 subunit of VLA-4 receptor)
integrin, alpha 5 (fibronectin receptor, alpha polypeptide)
integrin, alpha 6
integrin, alpha 7
integrin, alpha 8
integrin, alpha 9
integrin, alpha V (vitronectin receptor, alpha polypeptide, antigen CD51)
integrin, beta 1 (fibronectin receptor, beta polypeptide, antigen CD29 includes MDF2, MSK12)
integrin, beta 3 (platelet glycoprotein IIIa, antigen CD61)
integrin, beta 4
integrin, beta 5
integrin, beta 6
integrin, beta 7
integrin, beta 8
integrin-binding sialoprotein
laminin, alpha 1
laminin, alpha 2
laminin, alpha 3
laminin, alpha 4
laminin, alpha 5
laminin, beta 1
laminin, beta 2 (laminin S)
laminin, beta 3
laminin, beta 4
laminin, gamma 1 (formerly LAMB2)
laminin, gamma 2
laminin, gamma 3
reelin
secreted phosphoprotein 1
synaptic vesicle glycoprotein 2A
synaptic vesicle glycoprotein 2B; hypothetical protein LOC100128403
synaptic vesicle glycoprotein 2C
syndecan 1
syndecan 2
syndecan 3
syndecan 4
tenascin C
tenascin N
tenascin R (restrictin, janusin)
tenascin XB; tenascin XA pseudogene
thrombospondin 1
thrombospondin 2
thrombospondin 3
thrombospondin 4
vitronectin
von Willebrand factor

Show All List Genes

Show All Pathway Genes

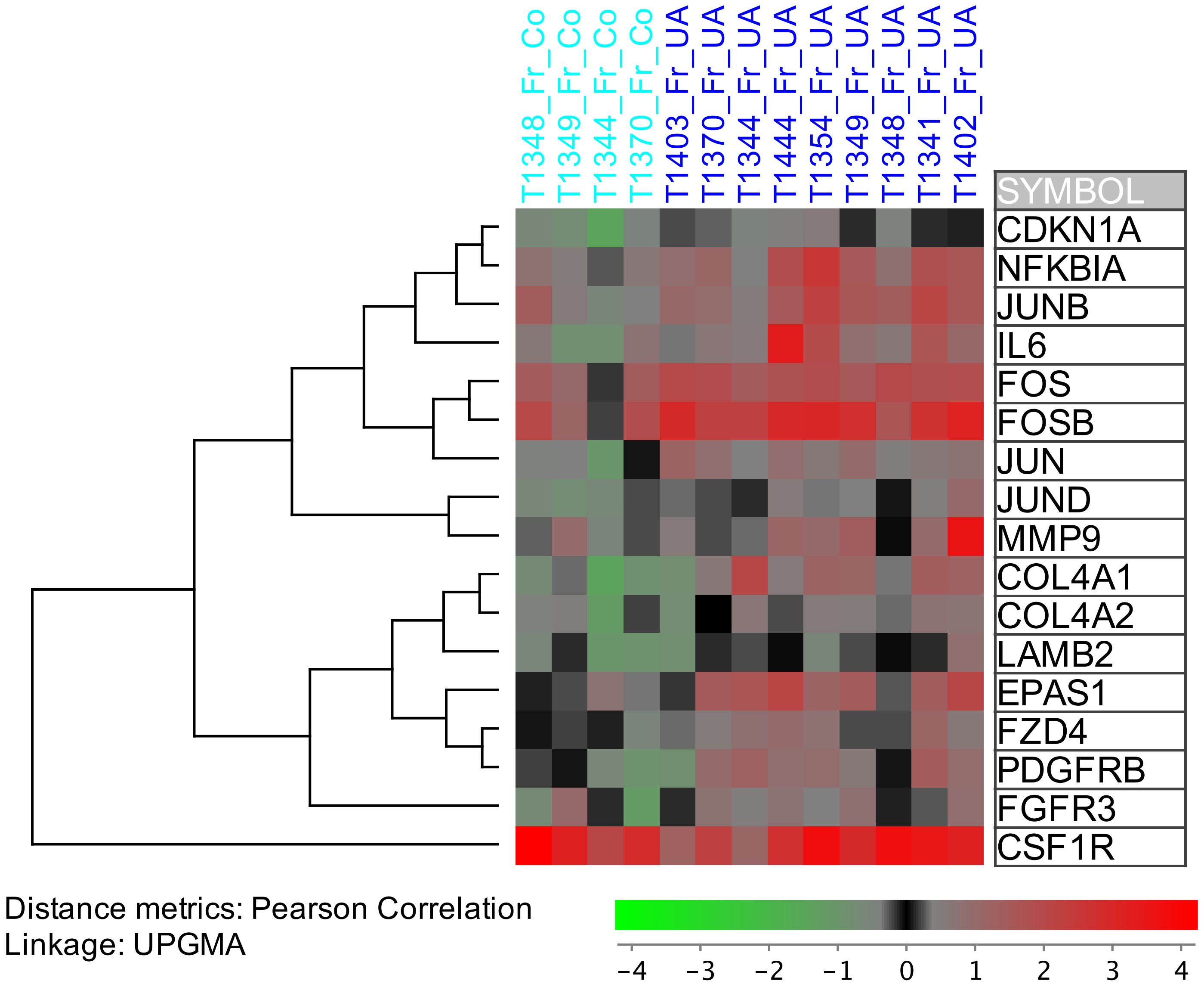
T1348_Fr_Co
T1349_Fr_Co
T1344_Fr_Co
T1370_Fr_Co
T1403_Fr_UA
T1370_Fr_UA
T1344_Fr_UA
T1444_Fr_UA
T1354_Fr_UA
T1349_Fr_UA
T1348_Fr_UA
T1341_Fr_UA
T1402_Fr_UA
SYMBOL
CDKN1A
NFKBIA
JUNB
IL6
FOS
FOSB
JUN
JUND
MMP9
COL4A1
COL4A2
LAMB2
EPAS1
FZD4
PDGFRB
FGFR3
CSF1R
Distance metrics: Pearson Correlation
Linkage: UPGMA
-4
-3
-2
-1
0
1
2
3
4

Supplementary Figure Legends

Figure S1. Hierarchical clustering (HCL) plot showing subtyping of the sphere- and fresh-cultures according to the 12-gene signature ([22]). Included in this analysis are: a) the new samples from this study (GEO set number pending), b) UA-samples from the previous analysis ([22]) and also the 178 core TCGA samples ([38]). The dendrogram colours to the left: red, blue and green specify GBM mesenchymal, neural/proneural and classical subtypes, respectively. The dendrogram colours on the top were assigned to the the 12-gene identifier signature according to the previously described method ([22]). Briefly, red, blue and green specify mesenchymal, neural/proneural and classical GBM subtypes, respectively. The expression values were log2 transformed.

Figure S2. HCL plot showing the expression of the differentially expressed genes between the groups (Fr-Co and Fr-UA). In this HCL only the 352 genes with the most variable expression between the groups Fr-Co and Fr-UA. Low expression is shown with green color while high expression is shown in red. Dendrogram colours light and dark blue specify core and UA samples respectively. The other dendrogram colours (to the left) indicate sets of genes up-regulated (magenta) and down-regulated in core samples (lemon colour). The expression values were log2 transformed.

Figure S3. Graphical representation of the notch signalling pathway according to the KEGG (Kyoto Encyclopedia of Genes and Genomes) database. The genes that are differentially regulated between the groups Fr-Co and Fr-UA are both labelled with red asterisks and listed below. The official gene symbols can be found in the Table S2 and the Figure 2D.

Figure S4. HCL plot showing the differential expression of the genes within the p53 signalling pathway between the groups Fr-Co and Fr-UA. Low expression is shown with green colour while high expression is shown in red. Dendrogram colours light and dark blue specify core and UA samples respectively. The expression values were log2 transformed.

Figure S5. Graphical representation of the p53 signalling pathway according to the KEGG (Kyoto Encyclopedia of Genes and Genomes) database. The genes that are differentially regulated between the groups Fr-Co and Fr-UA are both labelled with red asterisks and listed below. The official gene symbols can be found in the Table S2 and the Figure S4.

Figure S6. HCL plot showing the differential expression of the genes within the ECM-interaction signalling pathway between the groups Fr-Co and Fr-UA. Low expression is shown with green colour while high expression is shown in red. Dendrogram colours light and dark blue specify core and UA samples respectively. The expression values were log2 transformed.

Figure S7. Graphical representation of the ECM-interaction signalling pathway according to the KEGG (Kyoto Encyclopedia of Genes and Genomes) database. The genes that are differentially regulated between the groups Fr-Co and Fr-UA are both labelled with red asterisks and listed below. The official gene symbols can be found in the Table S2 and the Figure S6.

Figure S8. HCL plot showing the differential expression of the genes within the cancer signalling pathway between the groups Fr-Co and Fr-UA. Low expression is shown with green colour while high expression is shown in red. Dendrogram colours light and dark blue specify core and UA samples respectively. The expression values were log2 transformed.

Table S1. Patient samples characteristics.

UA-Sample	Tumor type	Core biopsy	Sphere culture success UA:Core	Growth pattern
**T1245**	WHO grade IV (GBM)	NO	NO	-
**T1246**	WHO grade II (Diffuse astrocytoma)	NO	NO	-
**T1251**	WHO grade IV (GBM)	NO	NO	-
**T1309**	WHO grade IV (reGBM)	NO	NO	-
**GBM25**	WHO grade IV (Gliosarcoma)	Yes	Yes	adherent
**T1311**	WHO grade IV (secGBM)	Yes	Yes:Yes	sphere
**T1336**	WHO grade IV (GBM)		NO	-
**T1341**	WHO grade IV (GBM)	Yes	Yes:Yes	sphere
**T1344**	WHO grade IV (GBM)	Yes	Yes:Yes	adherent
**T1348**	WHO grade IV (Giant cell GBM)	Yes	Yes:Yes	sphere
**T1349**	WHO grade IV (GBM)	Yes	Yes:Yes	sphere
**T1351**	WHO grade IV (GBM)	NO	NO	-
**T1354**	WHO grade IV (GBM)	NO	Yes	sphere
**T1370**	WHO grade IV (GBM)	Yes	Yes:Yes	adherent
**T1402**	WHO grade IV (GBM)	Yes	Yes:Yes	sphere
**T1403**	WHO grade IV (GBM)	NO	Yes	adherent
**T1406**	WHO grade II (Diffuse astrocytoma)	Yes	NO	-
**T1407**	WHO grade IV (reGBM)	Yes	Yes:Yes	sphere
**T1408**	WHO grade IV (GBM)	Yes	Yes:NO	sphere
**T1409**	WHO grade II (Diffuse astrocytoma)	NO	NO	-
**T1410**	WHO grade III (Oligo-astorcytoma)	NO	NO	-
**T1411**	WHO grade IV (GBM)	NO	NO	-
**T1412**	WHO grade IV (GBM)	Yes	NO	-
**T1413**	WHO grade IV (reGBM)	Yes	Yes:NO	sphere
**T1414**	WHO grade IV (GBM)	NO	NO	-
**T1415**	WHO grade IV (GBM)	NO	Yes	sphere
**T1416**	Metastasis	Yes	Yes:Yes	adherent
**T1417**	WHO grade IV (reGBM)	Yes	NO:NO	-

**T1418**	WHO grade I (Pilocytic astrocytoma)	NO	NO	-
**T1419**	WHO grade IV (GBM)	NO	Yes	sphere
**T1421**	WHO grade II (reEpendymoma)	NO	NO	-
**T1420**	WHO grade IV (reGBM)	NO	Yes	sphere
**T1422**	WHO grade IV (GBM)	Yes	Yes:NO	adherent
**T1424**	WHO grade IV (reGBM)	NO	Yes	sphere
**T1426**	WHO grade IV (GBM)	NO	NO	-
**T1427**	WHO grade I (rePilocytic Astrocytoma	NO	NO	-
**T1428**	WHO grade IV (GBM)	NO	Yes	sphere
**T1429**	WHO grade II (reEpendymoma)	NO	NO	-
**T1435**	WHO grade IV (reGBM)	Yes	Yes:Yes	sphere
**T1437**	WHO grade IV (secGBM)	NO	Yes	sphere
**T1444**	WHO grade IV (GBM)	NO	Yes	adherent
**T1445**	WHO grade IV (GBM)	NO	Yes	sphere

Abbreviations: WHO, world health organization; GBM, glioblastoma multiforme; reGBM, recurrent GBM; secGBM, secondary GBM.

Table S2 . The RNA integrity number (RIN) of total RNA extracted from UA-samples and tumor core biopsies.

Sample name	RIN (Sp-UA)	RIN (Sp-Core)	RIN (Fr-UA)	RIN (Fr-Core)
**T1311**	8.6	7.1	NA	NA
**T1341**	9.8	NA	9.9	NA
**T1344 ***	9.3	9.4	4	5.8
**T1348**	10	6.1	6.9	7.3
**T1349**	9	8.3	4	6.7
**T1354**	10	NA	9.6	NA
**T1370***	8.4	8.3	6.1	6.8
**T1402**	9.7	8.7	9.7	NA
**T1403***	9.4	NA	6.7	NA
**T1407**	9.4	6.7	NA	NA
**T1444***	9.1	NA	NA	NA
**Mean**	9.34	7.8	7.1	6.65
**SD**	1.19	0.53	2.43	0.63

Abbreviations: Sp, Sphere; UA, Ultrasonic aspiration; RIN, RNA integrity number; (*) growing Adherently in sphere condition.

Table S4 . FFPE Panel - PGM Ion Torrent of most known adult human gliomas, pediatric gliomas and neurofibromatoses or schwannomatosis

	Type	Name	Chromosome	Num Amplicons	Coverage
GLIOMAS	Gene CDS	**TP53**	chr17	30	98,8%
	Gene CDS	**EGFR**	chr7	88	99,8%
	Gene CDS	**PDGFRA**	chr4	70	100,0%
	Gene CDS	**PTEN**	chr10	29	100,0%
	Gene CDS	**PIK3CA**	chr3	65	98,5%
	Gene CDS	**PIK3R1**	chr5	50	99,5%
	Gene CDS	**RB1**	chr13	70	92,4%
	Gene CDS	**CDK4**	chr12	14	99,8%
	Gene CDS	**CDKN2A**	chr9	10	61,6%
	Gene CDS	**CDKN2B**	chr9	5	83,8%
	Genome region	**IDH1 (exon 4)**	chr2	5	100,0%
	Genome region	**IDH2 (exon 4)**	chr15	2	100,0%
	Genome region	**TERT promoter**	chr5	1	100,0%
	Gene CDS	**NOTCH1**	chr9	96	82,7%
	Genome region	**TACC3**	chr4	18	90,0%

	Genome region	**FGFR3**	chr4	20	75,9%
	Gene CDS	**FUBP**	chr1	54	98,2%
	Gene CDS	**CIC**	chr19	63	83,9%

PAEDIATRIC GLIOMA	Gene CDS	**ATRX**	chrX	134	98,8%
	Gene CDS	**ACVR1**	chr2	28	100,0%
	Gene CDS	**DAXX**	chr6	38	100,0%
	Gene CDS	**H3F3A**	chr1	9	70,6%
	Genome region	**KIAA1549**	chr7	24	86,6%
	Genome region	**BRAF**	chr7	31	96,5%

NEURO FIBROMATOSIS	Gene CDS	**NF1**	chr17	233	95,3%
	Gene CDS	**NF2**	chr22	89	83,7%
	Gene CDS	**SMARCB1**	chr22	26	100,0%
	Gene CDS	**SPRED1**	chr15	86	90,0%
	Gene CDS	**LZTR1**	chr22	57	92,2%

Table S7. Percentage of spheres formation from single cell of sphere cultures.

Sample name	UA-sample Passage	Sphere formation ratio In UA-sample	Core-sample Passage	Sphere formation ratio In Core-sample
**T1311**	P9	50%	P7	63%
**T1344 ***	P10	38%	NA	NA
**T1348**	P9	81%	NA	NA
**T1349**	P9	50%	P9	38%
**T1402**	P8	70%	P11	62%

Abbreviations: Sp, Sphere; UA, Ultrasonic aspiration; (*) growing Adherently in sphere condition.

Table S8. **Antibodies used for FACS, Immunocytochemistry, and Immunohistochemistry**

Directly conjugated antibodies	Producer	Primary Antibodies	Producer
CD133/2- APC	Miltenyi	alpha smooth muscle Actin (αSMA)	Abcam
CD133/2- PE	Miltenyi	Chitinase 3-like 1 (YKL40)	R & D Systems
CD56-FITC	Miltenyi	SOX9 (clone EPR12755)	Abcam
CD44-APC	eBioscinece	SOX2	Cell Signaling
CD15-APC	Miltenyi	Vimentin	R & D Systems
CD15-PE	Miltenyi	Anti-MAP2A	Millipore
CD34-APC	BD Pharmingen	Endosialin, clone B1/35	Millipore
CD146-APC	Miltenyi	CD271	Abcam
CD31-FITC	Miltenyi	O4, clone 81	Chemicon
CD271-APC	Miltenyi	hCD31	Abcam
CD166-PE	BD Pharmingen	hNestin	Abcam
CD9-FITC	eBioscinece	Ki67 (Clone SP6)	Abcam
Nestin-AF647	BD Pharmingen	PDGFB	Sigma Aldrich
GFAP-FITC	BD Pharmingen	PDGFR-B	Sigma Aldrich
CXCR4-PE	Miltenyi	PDGFR-α (D13C6)	Cell Signaling
		Doublecortin (c-18)	Santa Cruz
		S100 Antibody [8B10]	Abcam
		c-Kit	Abcam
		GFAP	Dako
		NeuN	Millipore

Secondary Antibodies used for FACS, Immunocytochemistry, and Immunohistochemistry.

**Secondary Antibodies**	**Producer**
Alexa Fluor 488 donkey anti-rabbit	Molecular Probes
Alexa Fluor 488 donkey anti-mouse	Molecular probes
Alexa Fluor 647 donkey anti-goat	Molecular probes
Alexa Fluor 647 donkey anti-Rabbit	Molecular probes
Alexa 647 donkey anti-mouse	Life Technologies
Alexa 594 Donkey anti-Goat	Molecular probes
Alexa 594 donkey anti-rabbit	Life Technologies

Supplementary method

Protocol

Preparing single cell suspension from ultrasonic aspiration of brain tumor sample

Materials:

- Ultrasonic aspirated specimen
- DPBS wo Ca^{2+} and $Mg2^{2+}$
- DMEM/f12 Medium
- Tryple (Life technology, cat # 12563011)
- DNase I
- FBS

Preparation of solutions:

- Washing solution DMEM/f12+4%FBS.
- DPBS wo Ca^{2+} and $Mg2^{2+}$ (50 ml)

Aliquoted in 50 ml tubes and kept +4 ºC.

Components<br><br>(Sphere culture medium)	Starting Concentration	Final Concentration
Hepes Buffer	1 M	10 mM
Pen/Strep	100X	1X
B27 w/o Vit A	50X	1X
EGF	20 µg/mL	20 ng/mL
bFGF	10 µg/mL	10 ng/mL
Heparin	0.5 mg/mL	2.5 µg/mL
DMEM/F12	1X	1X

Equipment:

- Sterile forceps
- Sterile blades
- 50 ml centrifuge tubes (BD)

- Petri dishes
- 50 ml syringes (to crush the sample instead of mortar)
- T-75 and T175 culture treated flasks for sphere and adherent culture.

Method:

1. Human brain tumor ultrasonic aspirated sample is aseptically collected at Oslo University Hospital, Rikshospital, the surgical theater, and transferred to the next building (on ice).
2. Handle the brain tumor sample under sterile conditions using a laminar airflow hood.
3. Pour the sample from the plastic collection bag into 500ml bottle carefully (2 bottles are need usually because of the big volume of aspirate)
4. Aliquot the sample into 50 ml tubes and keep the aliquots on ice while you are working .
5. Centrifuge at 1200rpm for 5min and aspirate the supernatant (be careful of aspirating the sample fragments which are precipitated with blood clot).
6. Add 1-2 ml medium and keep the sample on ice.
7. Mince the tissue into small fragments with blade, because the amount of the tissue fragments is too much, it was divided into 3 parts:
   Part 1) snap frozen in liquid nitrogen, part 2) mixed with FBS (1:1) and 10% DMSO and frozen in Mr.Frosty, Part3) to be continued with step 8
8. To generate single cell suspension, continue with processing the tissue mechanically through crushing the tissue fragments **gently then** collect them into a 50 ml tube and add washing solution to get easier filtration.
9. Filtrate the tissue through 100 µm filter with continuously adding for washing solution.
10. Add more DMEM/f12 (without serum) to the remaining tissue from first filtration (final volume with tissue 7 ml), add 7 ml tryple and DNase I (final conc. 10 ul/ml) and incubate at 37 ^{o}C, 5% CO_2 for 15min.
11. Add same volume washing solution to stop the enzymatic digestion, and filtrate again as in step 8.
12. Centrifuge at 1200rpm for 5 minutes at 4 ^{o}C and aspirate the supernatant. Add 1-3ml medium and keep the pellet at 4 ^{o}C.

13. Add 10 ml of red blood lysis buffer (BD pharm Lyse) and incubate for 5 minutes at RT, stop the reaction by adding NH medium or serum, and Centrifuge at 1200rpm for 5 min. removed the supernatant and resuspend the cell pellet

a. For cell culture, resuspend the pellet in appropriate sphere culture medium (5-10 ml).
b. For FACS or MACS resuspend pellet in recommended buffer.

14. Count cell number by diluting the cell suspension with DMEM (1:20 or 1:40).
15. Check the cell viability by taking 100µl of the sample, 50µ/eppendrof
16. cell counting and viability assessment (by cell counter):

Dead cells:

Total cells:

Viable cells: total – dead= /ml

Viability: =vible/total%

17. Clean the hood and working area with water first and then 70% Ethanol, and UV.

Comments:

- Hard crushing could affect the viability, for that we recommend gentle crushing.

-Viability after RBC lysis buffer is improved
